# Examining toxic trace element exposure in American alligators

**DOI:** 10.1016/j.envint.2019.04.035

**Published:** 2019-05-08

**Authors:** Frances M. Nilsen, John A. Bowden, Thomas R. Rainwater, Arnold M. Brunell, Brittany L. Kassim, Phil M. Wilkinson, Louis J. Guillette, Stephen E. Long, Tracey B. Schock

**Affiliations:** a National Institute of Standards and Technology, Chemical Sciences Division, Hollings Marine Laboratory, Charleston, SC, USA; b Medical University of South Carolina, Marine Bio-medicine and Environmental Science Program, Charleston, SC, USA; c Center for Environmental and Human Toxicology, Department of Physiological Sciences, College of Veterinary Medicine, University of Florida, Gainesville, FL, USA; d Baruch Institute of Coastal Ecology and Forest Science, Clemson University, P.O. Box 596, Georgetown, SC, USA; e Tom Yawkey Wildlife Center, South Carolina Department of Natural Resources, 1 Yawkey Way South, Georgetown, SC, USA; f Florida Fish & Wildlife Conservation Commission, Eustis, FL, USA

## Abstract

Toxic trace element exposure occurs through release of the ubiquitous and naturally occurring elements arsenic (As), cadmium (Cd), lead (Pb), and mercury (Hg). The unique environmental conditions of the wetland ecosystems along the southeastern Atlantic coast of the United States lead to the accumulation of Hg which is greater than in most other ecosystems in the country. There are also point sources of As, Cd, and Pb in this region. To effectively monitor trace element concentrations, and consequently the potential human exposure, accessible local sentinel species are needed. In this study, concentrations of As, Cd, Pb, Hg and six other trace elements (Al, Ni, Cu, Zn, Se, Mo) were examined in American alligators (*Alligator mississippiensis*) from seven wetland sites in South Carolina and Florida and assessed for their utility as a sentinel species for human trace element exposure. Alligators were chosen as a potential sentinel as they share a common exposure with the local human population through their aquatic diet, and they are directly consumed commercially and through recreation hunting in this region. Sex was significantly related to the concentration of Zn, Mo, and Al, but not As, Pb, Hg, Cd, Se, or Cu. Site specific differences in element concentrations were observed for As, Pb, Hg, Cd, Se, Zn, and Mo. Size/age was significantly related to the element Hg and Pb concentrations observed. The observed concentration ranges for the four toxic elements, As (6–156 ng/g), Cd (0.3–1.3 ng/g), Pb (3–4872 ng/g), and Hg (39–2765 ng/g), were comparable to those previously reported in diverse human populations. In this region alligators are hunted recreationally and consumed by the local community, making them a vehicle of direct human toxic element exposure. We propose that the similarity in As, Cd, Pb, and Hg concentrations between alligators observed in this study and humans underscores how alligators can serve as a useful sentinel species for toxic element exposure.

## 1. Introduction

Trace elements are naturally occurring and ubiquitous in “trace” concentrations below 10 ng/g throughout the Earth’s crust and soil (Shia et al., 1999; UNEP, 2013; Pirrone et al., 2010; Tchounwou et al., 2012). Environmental trace element concentrations increase due to anthropogenic activities, such as mining, industrial manufacturing, and medical waste incineration (Tchounwou et al., 2012). The elements cadmium (Cd), lead (Pb), arsenic (As), and mercury (Hg) have no known beneficial or nutritional value for humans and are potentially toxic (Goyer, 2004). These toxic elements are known to cause systemic organ damage and are classified as either known or possible human carcinogens making them a public health concern (Goyer, 2004; Boffetta, 1993).

Human exposure to the toxic elements is often monitored using U.S. National Health and Nutrition Examination Survey (NHANES) data since it is representative of the diverse U.S. population. While there is more than one route of exposure (i.e. ingestion, inhalation, dermal contact) there are low concentrations of the toxic trace elements in U.S. food sources, according to the Total Diet Study (TDS) conducted by the Food and Drug Administration (Egan et al., 2002). Consumption of wild caught fish and other seafood is related to greater Hg, Cd, and As exposure (Awata et al., 2016; Xue et al., 2012; Mahaffey et al., 2009; Ju et al., 2012). Coastal populations of the U.S. consume more fish and seafood than inland populations, specifically longer-lived sport fish that are known to have greater contaminant concentrations (Mahaffey et al., 2009).

The coastal wetlands of the southeastern U. S. are a known area of high Hg contamination due to atmospheric deposition, local point sources, the hydroperiod, humidity, and soil composition of this region (Julian, 2013; Chasar et al., 2009; Chen and Folt, 2000; Mason et al., 1995). When Hg concentrations in largemouth bass *(Micropterus salmoides)* were identified as hazardous to human consumers, measures were taken to reduce the anthropogenic output of Hg across the southeast (Adams et al., 2003; Axelrad, 2011; Bryan et al., 2007; Burger et al., 2000; Cleckner et al., 1998; Eisemann et al., 1997; Heaton-Jones et al., 1997; Horai et al., 2014; Hord et al., 1990; Julian, 2013; Lange et al., 1994, 2000; Rumbold et al., 2001, 2002). However, there are still a wide range of industrial manufacturing facilities throughout the region that are point sources for the toxic trace elements. These point sources are the battery manufactories in Florida that are anthropogenic sources of Cd and Pb; the coal-fired power plants in South Carolina that emit Hg; and the wood preservation chemicals used commercially throughout the region that frequently contain As (Tchounwou et al., 2012). The variety of toxic element inputs to this region lead to the local predatory wildlife experiencing greater trace element exposure through their diet than in other locations (Hord et al., 1990; Rumbold et al., 2002; Kannan et al., 1998; Khan and Tansel, 2000; Rey et al., 2012; Nilsen et al., 2016).

Effective monitoring of new and ever-changing environmental and human health hazards requires diverse wildlife indicator species. It has recently been proposed that emerging sentinel species should signal more than a “cause and effect” relationship specific to the organism (Tabor and Aguirre, 2004). Instead the chosen species should highlight ecological relationships between an impacted sentinel and its ecosystem caused by the contamination that are relevant to the well-being of local human populations (Tabor and Aguirre, 2004).

The American alligator *(Alligator mississippiensis)* is a keystone predator that is critical to the balanced structure and function of wetlands throughout the southeastern U.S., directly connecting alligators to the health of the wetland ecosystem (Palmer and Mazzotti, 2004; Mazzotti et al., 2009; Bondavalli and Ulanowicz, 1999; Kushlan, 1974; Rosenblatt and Heithaus, 2011). The toxic trace elements have continually been reported in the tissues of alligators sampled across Florida and South Carolina (Nilsen et al., 2017b; Burger et al., 2000; Horai et al., 2014; Delany et al., 1988; Campbell et al., 2010; Yanochko et al., 1997). The long lifespan, broad diet, and relatively strong site fidelity have allowed alligators to be used in monitoring the effects of contaminants both at the population and individual levels for decades (Nilsen et al., 2017b; Wilkinson et al., 2016; Hord et al., 1990; Lange et al., 2000; Delany et al., 1988; Milnes and Guillette, 2008; Guillette et al., 2000; Gunderson et al., 2003, 2004, 2006; Hamlin et al., 2010; Elsey et al., 2008). The alligator has a varied diet consisting of both vertebrate and invertebrates but is comprised mostly of fish (Rice, 2004; Rice et al., 2007; Delany et al., 1999). The fish species consumed overlap with the sport-fish consumed by humans, as well as the forage/bait fish of the lower trophic levels (Rice, 2004; Rice et al., 2007). The similar diets of alligators and humans in this region make alligators a worthy candidate to observe toxic element concentrations that humans may be exposed to via fish consumption (Rice et al., 2007). Additionally, it is common in the southeastern United States for the routine consumption of alligator meat via both commercial sales and recreational hunting (Rice et al., 2007; Delany et al., 1999; FFWCC, 2015; Tipton et al., 2017a, 2017b; Smith et al., 2017). Reptiles are cold-blooded and have a slower metabolism than mammals, which often raises concerns regarding their legitimacy as a sentinel for humans. However, their slow metabolism leads reptiles to be more robust when exposed to metal concentrations that are documented to be toxic to birds and mammals (Grillitsch and Schiesari, 2010; Crain and Guillette, 1998; Nagy et al., 1999; Weir et al., 2010). This unique ability may provide the opportunity to better understand the onset of effects that are detrimental to human health, but additional comparative studies are needed to improve the paucity of existing data (Grillitsch and Schiesari, 2010; Crain and Guillette, 1998; Nagy et al., 1999; Weir et al., 2010).

In this study, 17 trace element concentrations were examined in alligator blood samples from seven sites in the southeastern U.S.; five in Florida, and two in South Carolina. Ten trace elements were of measurable quality and were used in the analysis. Each site had different environmental and anthropogenic circumstances for comparison of accumulative trace elements in a predator experiencing chronic exposures. Using the measured trace element concentrations, we evaluate the utility of the American alligator as a sentinel species for local toxic trace element exposure.

## 2. Methods

For this study, adult alligators were captured, sampled, and released back into their natural environment. Blood samples were analyzed for a suite of trace elements using high quality analytical methods. The observed concentrations of trace elements were statistically compared between study sites. Each methodical step is described in detail below.

### 2.1. Study area & sample collection

**Fig. 1.**
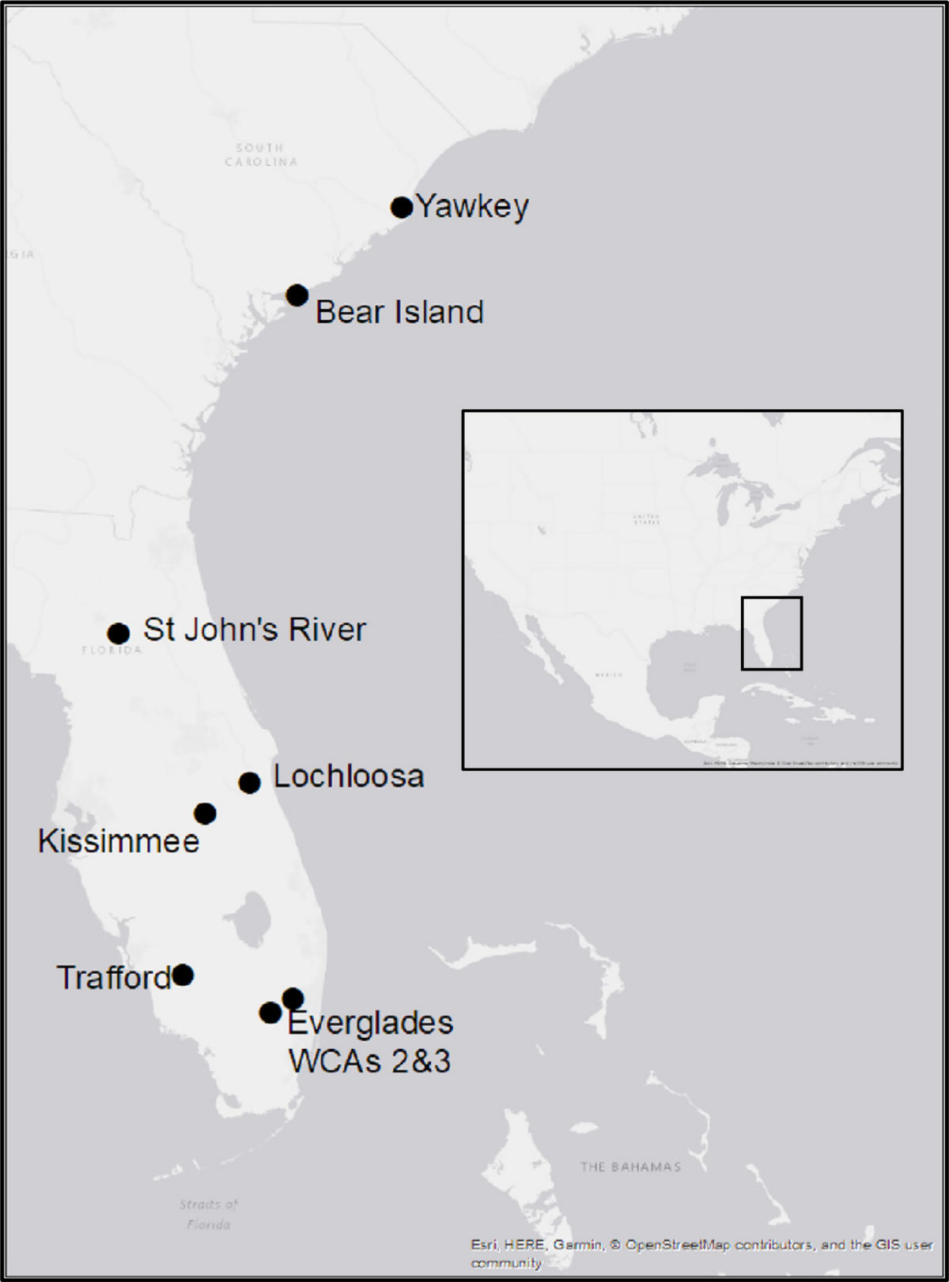
Map of sites where American alligator (*Alligator mississippiensis*) blood samples were collected for this study, made using ArcMap10 (ESRI, Redlands, CA, USA).

Alligators were sampled opportunistically at seven sites along the southeastern Atlantic U.S, two in South Carolina, and five in Florida between March 28 and June 30, in the spring of 2011, 2012, and 2014 (Table S4). Each site is associated with different types of anthropogenic influences and therefore potentially different environmental trace element profiles (Fig. 1). The two sites in South Carolina currently have no trace metal values reported for alligators in the literature. The two sites were the Tom Yawkey Wildlife Preserve, which is adjacent to a paper mill, steel mill, and the largest coal-fired power plant in South Carolina; and Bear Island Wildlife Management Area, which is located within the undeveloped Ashepoo, Combahee, and Edisto (ACE) river basin estuary and has been considered a reference site for previous ecotoxicological evaluations (Bangma et al., 2017; Cobb et al., 2002). The five sites from Florida have been the object of many Hg monitoring studies in the past, but few have examined other trace elements (Nilsen et al., 2016, 2017a; Burger et al., 2000; Heaton-Jones et al., 1997; Horai et al., 2014; Lange et al., 2000; Delany et al., 1988). Historically, Lake Lochloosa, and the St. John’s River are low Hg sites, lakes Kissimmee and Trafford are moderate Hg sites, and the Everglades is a site of high Hg contamination due to its natural biogeochemistry (Rumbold et al., 2001, 2002; Rey et al., 2012; Nilsen et al., 2016; Krabbenhoft et al., 1998; Julian, 2013).

American alligators were handled by researchers with the Florida Fish and Wildlife Conservation Commission, the South Carolina Department of Natural Resources, and the Medical University of South Carolina using methods approved by the American Society of Ichthyologists and Herpetologists (Beaupre et al., 2004). Animals were identified by sex and classified as adults based on a measure of body length, which is determined using the snout to vent length (SVL). Adults were identified as having an SVL ≥ 80 cm, this cutoff measurement was established in a previous report in Florida pertaining to reproductive maturity, and the availability of alligators at Bear Island (Woodward et al., 1992). Morphometric data for the sampled animals are reported in Tables 1 and S4.

Whole blood samples were collected following the method described by Myburgh et al. (2014). Briefly, the samples were collected at the time of capture from the post-occipital venous sinus with 2.5 cm 18.5-gauge needles (Lot # 305196, BD, Franklin Lakes, NJ, USA) and 60 mL luer lock syringes (Lot # 09F058B, BD). Samples were transferred from the syringe to lithium-heparin blood collection tubes (Lot # 1178410; 0246555, BD). Blood samples were stored on ice for no > 5 h before being frozen at −80 °C until analysis.

### 2.2. Chemicals

Unless otherwise mentioned, all materials were obtained from the Office of Reference Materials at the National Institute of Standards and Technology (NIST) (Gaithersburg, MD, USA), including the Standard Reference Material (SRM) 3100 series of primary standard solutions utilized in inductively coupled plasma mass spectrometric (ICP-MS) analysis (Lot information provided in Table S1). Control materials utilized included SRM 955c Toxic Metals in Caprine Blood (NIST), and Seronorm, Trace Elements in Whole Blood (Sero AS, Billingstad, Norway; Lot #1112691). Nitric acid (Optima Grade, 67–70%), hydrochloric acid (Optima Grade, 32–35%) (Fisher Scientific, Waltham, MA, USA), and deionized 18 MQ cm water (Millipore EMD, Darmstadt, Germany) were used in all experiments.

### 2.3. Sample preparation for elemental analyses

Blood samples were thawed to room temperature, and gently rocked to mix the contents prior to analysis. Approximately 0.5 g of blood was transferred into a pre-cleaned Teflon microwave digestion vessel (CEM Corporation, Matthews, NC, USA), and gravimetrically weighed. All samples were treated identically in the digestion procedure. Internal standards consisting of Scandium (Sc), Yttrium (Y), and Ruthenium (Ru) (250 ng) dissolved in acidified aqueous solutions were weighed by difference into the digestion vessel, followed by addition of approximately 3.5 mL of nitric acid. Digestion was conducted using a CEM Microwave Accelerate Reaction System (MARS) Xpress (Matthews, NC) set to ramp for 10 min to 125 °C, hold for 5 min, then ramp for 5 min to 210 °C with a hold of 15 min, followed by a 15 m in cool down. The resulting digest was transferred to a nitric acid rinsed 50 mL centrifuge tube and diluted to approximately 50 g, with 1.5 mL hydrochloric acid added for element stabilization. The Seronorm control material replicates (*n* = 12) were weighed, digested, and analyzed under the same conditions.

All samples were measured on a Thermo X2 quadrupole inductively coupled plasma mass spectrometer (ICP-MS, Thermo Fisher Scientific, Waltham, MA, USA), equipped with a standard sample introduction system and an ESI SC4 auto sampler (Elemental Scientific, Omaha, NE, USA). The ICP-MS was operated in kinetic energy discrimination mode with a collision gas of 1% ammonia in balance helium, operating at 3.0 mL/min or 8% H_2_ in balance helium, operating at 4.0 mL/min. The addition of collision gas serves to reduce plasma and matrix-based interferences as well as decreasing the transmission of low kinetic energy polyatomic ions. The method utilized peak jumping, with each of five replicate runs consisting of 50 sweeps, and a dwell time of 25 ms. The data obtained from monitoring aluminum (Al), scandium (Sc), vanadium (V), chromium (Cr), manganese (Mn), cobalt (Co), nickel (Ni), copper (Cu), zinc (Zn), arsenic (As), selenium (Se), rubidium (Rb), strontium (Sr), yttrium (Y), molybdenum (Mo), ruthenium (Ru), cadmium (Cd), tin (Sn), and lead (Pb) were exported as a CSV file to Microsoft Excel (Microsoft Corporation, Redmond, WA, USA) spreadsheet for off-line calculations.

Standard solutions were prepared from NIST SRM 3100 series primary elemental solutions (Table S1). The working calibration stock solutions were prepared by gravimetric dilution and external ten-point calibration curves were constructed ranging from 0.5 ng/g to 1000 ng/g. Individual elemental curves were defined by the data set, resulting in a five-point calibration curve within the sample range. Analytical signals were corrected for blank contributions. A first order fit was applied to the data, where the slope and intercept from the calibration curves were based on the measured isotopic responses from SRM calibration solutions and utilized to calculate the concentration of trace elements in the samples.

### 2.4. Sample preparation for mercury analysis

Blood samples were thawed to room temperature, and gently rocked to mix the contents prior to analysis. Approximately 100 mg of alligator whole blood was pipetted into a nickel weigh boat and analyzed by direct combustion atomic absorption spectroscopy using a direct mercury analyzer DMA-80 (Milestone Scientific, Shelton, CT). NIST SRM 3133, Mercury Standard Solution was utilized for external calibration and SRM 955c Levels 2 (*n* = 16) and 3 (*n* = 45) was used as a control material for the Hg measurements. The control material was treated identically to the samples. The absorption data was exported from the DMA-80 as a CSV file to Microsoft Excel for off-line calculations.

### 2.5. Quality control measures

**Fig. 2.**
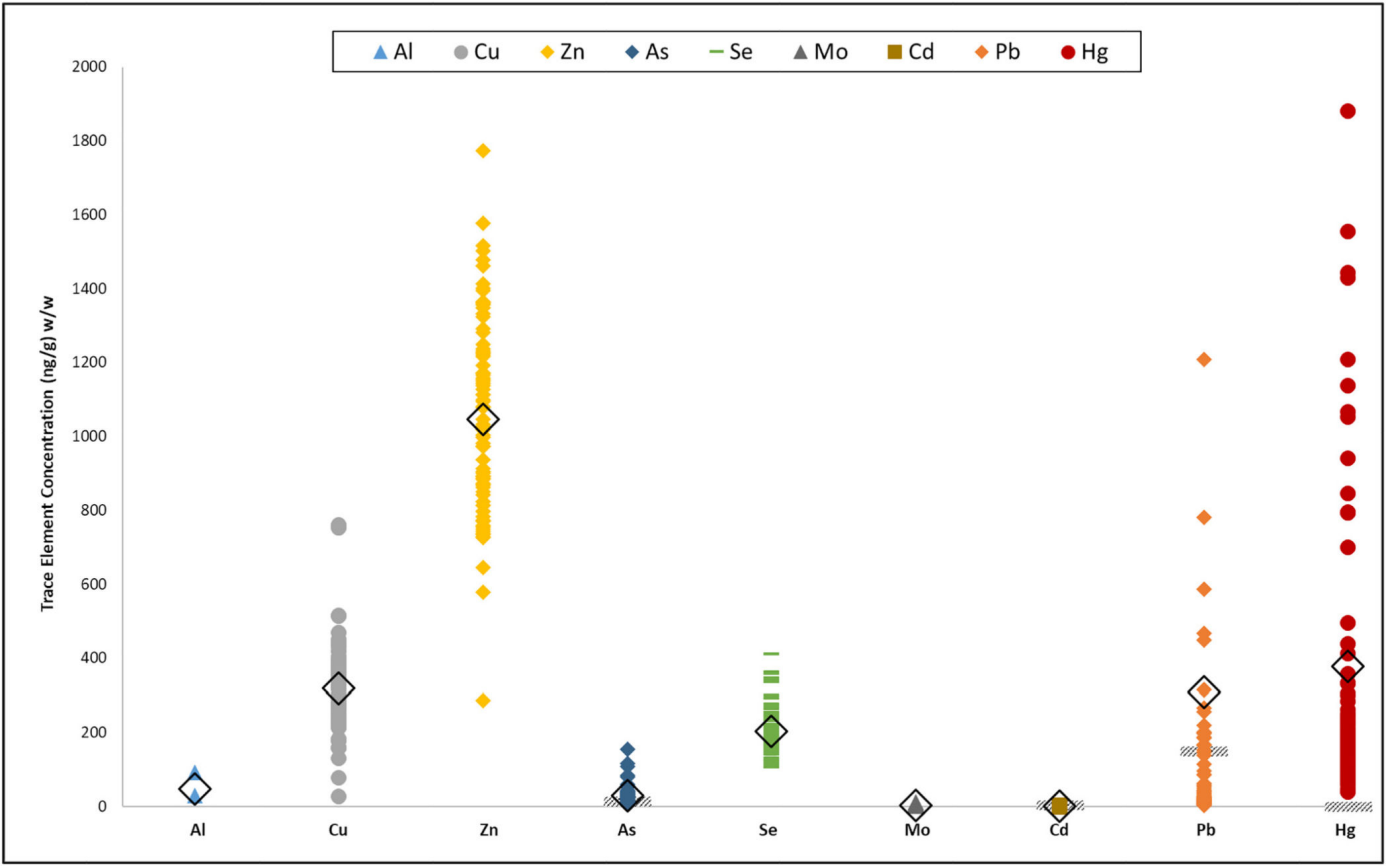
The range of trace element concentrations measured in the American alligator (*Alligator mississippiensis*) blood samples collected from South Carolina and Florida used in this study. The mean of all values is denoted with the open diamond, human health effects for the toxic trace elements are denoted with diagonal lines.

**Fig. 3.**
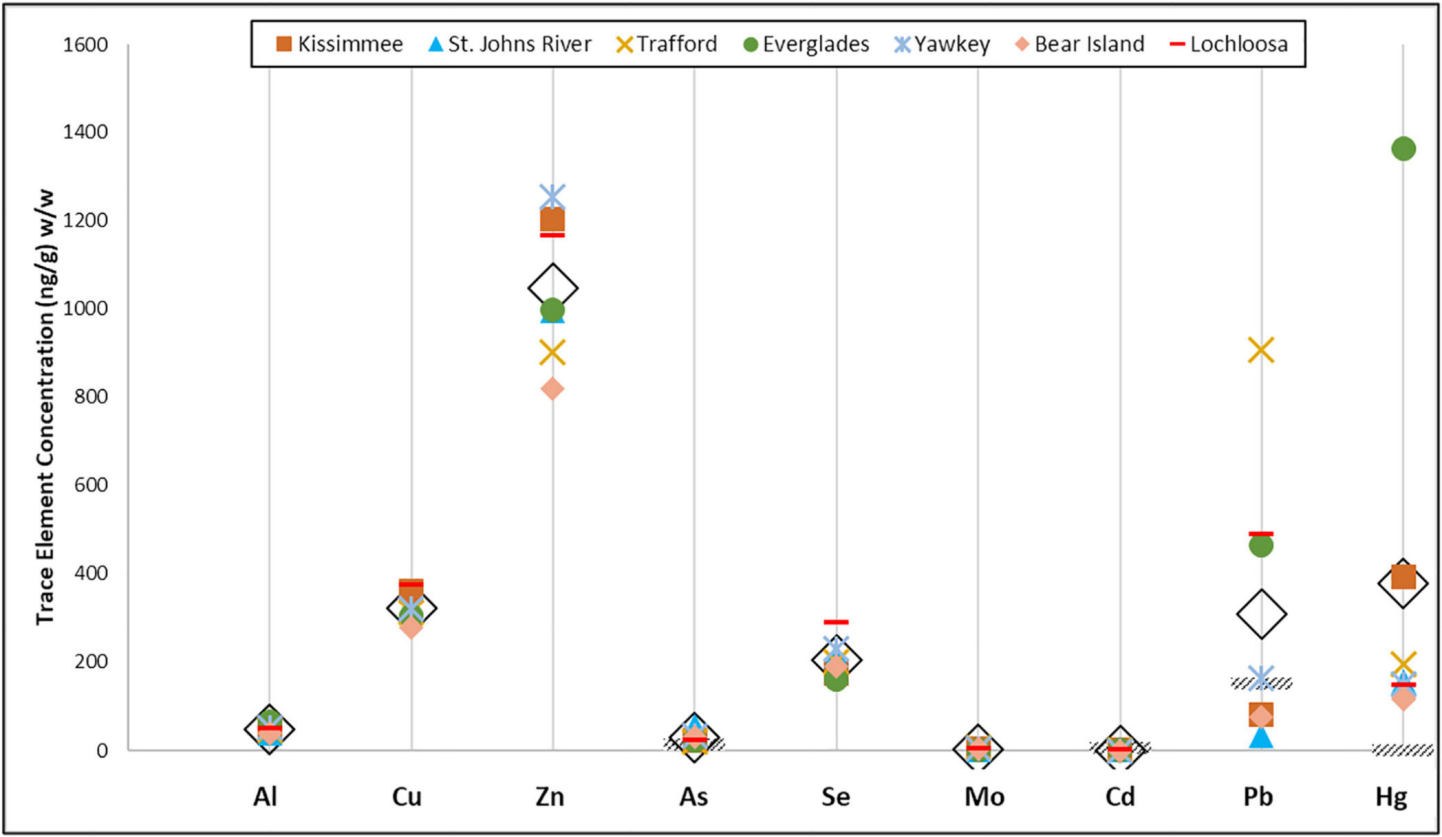
The mean trace element concentrations measured in the American alligator (*Alligator mississippiensis*) blood samples at each site in South Carolina and Florida used in this study. The mean of all values is denoted with the open diamond, human health effects for the toxic trace elements are denoted with diagonal lines.

To assess measurement quality and technical repeatability of the elemental concentrations detected in the alligator blood samples, the reference materials, Seronorm Trace Elements in Whole Blood L-3 (Lot# 1112691; SERO, ALS Scandinavia AB, Lulea, Sweden), and NIST SRM 955c Toxic Elements in Caprine Blood levels 2 and 3, were used. The measured values agreed with the certified values provided by the manufacturers for all elements, except for V, Rb, and Sr (Tables S2 & S3). The measured value %RSD for V, Rb, and Sr were greater than the certified %RSD (V: 33% and 19%, respectively; Rb: 4% and 1%, respectively; Sr: 12% and 1%, respectively) and were excluded from the analysis to ensure reporting of quality measurements. Of the 17 trace elements measured in the alligator blood samples, ten were accurately detected above the limit of detection and ensured with replicable control materials (Al, Ni, Cu, Zn, As, Se, Mo, Cd, Pb, Hg). All elements, except Ni, and Cd were detected at all seven sites (Table 1, Figs. 2 & 3). Ni was detected in only 15 samples and it was excluded from the statistical analyses, but the measurements are reported in Table S4, with the limits of detection and quantification.

### 2.6. Statistical analysis

All statistical analyses were conducted using SAS 9.4 (Cary, NC, USA). Descriptive statistics (mean, standard deviation, range) were calculated prior to testing for the assumptions of parametric statistics and are reported in Table 1. The elements that were found to be above the limit of detection did not demonstrate a normal distribution or equal variances across the population of alligators sampled. A log_10_ transformation did not improve either assumption for all elements. The SVL measurements met the assumptions for each location, but not for sex which is not surprising as male alligators are larger than females. Since the assumptions of parametric statistics were not met for all data, and the data is left-censored by the limit of detection (LOD) of the instrumentation, a non-parametric regression model using the maximum likelihood estimation (MLE) was used. The MLE model (SAS*proc LifeReg)* provided the ability to include left-censored data and was used to determine which variables contributed to the trace element concentrations observed (Helsel, 2005). *Location, Sex,* and *SVL* were tested individually, season and year were not tested as all samples were collected within the same 12-week period annually, and different sites were sampled each year. The combined term *Sex* + *SVL* was not included as the parameters co-vary because alligators are a sexually dimorphic species where males grow larger than females (Wilkinson et al., 2016). SVL is also used as a proxy for age in this species (Wilkinson et al., 2016). Sex and SVL are confounding factors in this study, since the sex ratio was not equal overall, or at any of the seven study sites (Table 1). Likewise, *SVL* + *Location* and *Sex* + *Location* were not included due to the uneven sex ratios across all study locations and the stratification of sexes by SVL observed at most but not all sites. The alligators were captured opportunistically, and sex was determined upon capture. The sex-ratio of the collected alligators was uneven, but as a previous study we conducted observed no sex-based differences in Hg concentration, we proceeded with the current investigation (Nilsen et al., 2016). Least squares mean difference comparison (LS means) with a Tukey-Kramer post-hoc correction was used for pairwise comparisons of *Location*, and *Sex* for each trace element. All SAS syntax that was used to conduct this analysis is provided in the Supplemental information.

## 3. Results

### 3.1. Trace element concentrations

The toxic elements (As, Pb, Hg, Cd), essential elements (Se, Zn), and transition metals (Cu, Mo, Al) were observed in alligators at all study sites (Tables 1 & S4). The highest mean concentrations of each trace element were not all observed at the same site, which suggests regional differences in contaminant inputs and accumulation in alligators. The alligators from Yawkey Wildlife Center had the highest mean concentrations of Zn (1255 ± 228 ng/g, Table 1). The Lake Lochloosa alligators had the highest mean concentration of Cu (372 ± 81 ng/g). St. John’s River alligators had the highest mean concentration of As (57 ± 50 ng/g). The alligators from Lake Trafford had the highest mean concentration of Mo (7 ± 2 ng/g) and Pb (906 ± 73 ng/g). The Everglades had the highest mean concentration of Hg (1364 ± 673 ng/g). The range of mean concentrations of each element in alligators across all sites varied widely, with the ranges of some elements (Mo, and Cd) spanning only a few nanograms, to ranges spanning an order of magnitude (Hg) (Tables 1 & S4; Figs. 2 & 3).

### 3.2. Influence of Sex & SVL on element concentrations

The trace elements that were significantly influenced by *Sex* and/or *SVL* in the MLE model were Zn, Mo, Al, Pb, and Hg (Table S5). The elements that were not significantly influenced by *Sex* and/or *SVL* were As, Cd, Se, and Cu (Table S5).

#### 3.2.1. Toxic elements

The mean Hg and Pb concentrations observed were not influenced by *Sex* but were influenced by *SVL*, which is indicative of age (MLE *p* < 0.0001, *p* = 0.0001, respectively, Table S5). As such, larger and older alligators will naturally have bioaccumulated more Hg and Pb than younger smaller alligators (Chasar et al., 2009; Mason et al., 1995; Chen and Folt, 2000).

To better understand the relationship between *Sex, SVL,* and toxic element concentrations in alligators, the Pb, Hg, As, and Cd measurements were statistically examined for a potential confounding relationship that could limit their use as a sentinel species. When each of the toxic metals were plotted by *Sex* and *SVL,* no apparent relationship appeared (Fig. S1). Interestingly, the Pb and Hg measurements had several individuals with higher concentrations than the other alligators. In the case of Pb, there were four males that had nearly double the Pb concentration of the rest of the alligators sampled. When these samples were removed from the plot, there was still no significant relationship between *Sex, SVL* and Pb concentration. The high Hg concentrations observed in the Everglades, which is a known site of Hg contamination, are not anomalous as we speculate the Pb concentrations may have been. Several mid-size males from the Everglades have the greatest observed Hg concentrations (Fig. S1). This relationship was recently reported for alligator populations at sites in Florida and South Carolina (Lawson et al. *Unpublished data).* However, when the alligators from the Everglades are removed, this relationship disappears (Fig. S1).

#### 3.2.2. Essential and transition elements

The only essential element concentration in alligators to be influenced by a co-varying factor was Zn. The observed Zn concentrations in alligators at the study sites were not influenced by *SVL* but were influenced by *Sex* (MLE *p* < 0.0001, Table S5). The transition metals Mo and Al were observed in concentrations in alligators that were significantly influenced by *Sex* (MLE *p* values = 0.03, Table S5) and *SVL* (*p* values ≤ 0.04).

### 3.3. Influence of Location on element concentrations

*Location* significantly influenced the concentration of the toxic (As, Pb, Hg, Cd), essential (Se, Zn), and transition (Mo, Al) elements in the MLE model. The site-specific differences between the element concentrations observed in alligators were evaluated by the LS means comparison.

#### 3.3.1. Toxic elements

*Location* significantly contributed to the As concentration in the alligators sampled at St. John’s River (mean As = 57ng/g ± 50ng/g, Table 1; MLE *p* < 0.0001, Table S5), the Everglades (mean As = 24 ng/g ± 7 ng/g; *p* = 0.01), Lake Kissimmee (mean As = 23 ng/g ± 5 ng/g; *p* = 0.002), Lake Lochloosa (mean As = 23 ng/g ± 7 ng/g; *p* = 0.004), and Lake Trafford (mean As = 22 ng/g ± 6 ng/g; *p* = 0.004). The LS mean difference comparison elucidated statistical differences between the alligators with the greatest mean As concentration (57 ± 50 ng/g) from St. John’s River and all other sites (*p* values ≤ 0.0005, Table S6). There was also a statistical difference between the As concentration in alligators from Lake Kissimmee and Yawkey, even though the range of the As concentrations in these alligators overlapped between sites (*p* = 0.04, Tables 1 and S6).

*Location* significantly influenced the Pb concentration in alligators at Lake Trafford, where the greatest mean Pb concentration in alligators was observed (mean Pb = 906 ng/g ± 73 ng/g MLE *p* = 0.03, Tables 1 & S5). The LS mean difference comparison revealed a significant difference between the Pb concentrations in alligators from Lake Trafford and St. John’s River (mean Pb = 33 ± 48 ng/g), which had the highest and lowest mean Pb concentrations observed in alligators in this study (*p* = 0.04, Table S6).

*Location* was influential to the Hg concentrations observed in alligators at the Everglades site (*p* < 0.0001; mean Hg = 1364ng/g ± 673 ng/g, Table 1), Lake Kissimmee (*p* < 0.0001; mean Hg = 393 ng/g ± 204 ng/g), and Lake Trafford (*p* = 0.004; mean Hg = 194 ng/g ± 73 ng/g), which were the three sites with the greatest mean Hg concentrations. The LS comparison highlighted the Hg concentrations observed in Everglades alligators as being highly significantly different from Hg concentrations in alligators at all other sites (*p* values < 0.0001, Table S6). Lakes Kissimmee and Trafford alligator Hg concentrations were statistically different from the alligators at the four sites that had lower mean Hg concentrations observed (Bear Island 118 ng/g ± 58 ng/g, Lochloosa 146 ng/g ± 67 ng/g, St. John’s River 153 ng/g ± 49 ng/g, and Yawkey 150 ng/g ± 49 ng/g; *p* values ≥ 0.05, Tables 1 & S6).

*Location* was a significant influence on Cd concentrations observed in alligators at all study sites except Bear Island (mean Cd = 0.7 ng/g ± 0.01 ng/g, Table 1) and Yawkey (mean Cd = 0.7 ng/g ± 0.2 ng/g), which had the lowest mean Cd concentrations (*p* values < 0.0001, Table S5). Bear Island and Yawkey alligators were also significantly different in mean Cd concentration from alligator Cd concentrations at all other sites (LS *p* values > 0.03, Table S6). The LS comparison also showed that the Cd concentrations observed in alligators from the Everglades (mean Cd = 1.1 ± 0.4 ng/g) were significantly different from the concentrations observed in alligators from Lake Kissimmee (mean Cd = 1.0 ± 0.01 ng/g; LS *p* = 0.0007), Lake Lochloosa (mead Cd = 1.0 ± 0.2 ng/g; *p* = 0.01), and Yawkey (*p* < 0.0001).

#### 3.3.2. Essential elements

The Se concentrations observed in alligators were influenced by *Location* at the Everglades site (mean Se = 160 ng/g ± 28 ng/g; *p* < 0.0001), Lake Kissimmee (mean Se = 174 ng/g ± 24 ng/g; *p* = 0.0006), Lake Lochloosa (mean Se = 289 ng/g ± 54 ng/g; *p* = 0.004), and St. John’s River (mean Se = 213 ng/g ± 107 ng/g; *p* = 0.01), which do not appear to be different from the other three sites based on their means and ranges (Table 1). The LS comparison revealed that these four sites have differences within their mean Se concentrations as compared to the other sites. The concentrations observed in alligators at Lake Lochloosa and St. John’s River are statistically different from those in alligators at the Everglades, Lake Kissimmee, Bear Island, and Lake Trafford sites (*p* values < 0.0008, Table S6). The Se concentration observed in alligators at Lake Kissimmee was also significantly different from the Se concentrations observed in alligators from Yawkey (*p* = 0.01).

*Location* was influential to the alligators at Bear Island which had the lowest mean Zn concentration observed in alligators (819 ng/g ± 41 ng/g, *p* = 0.0007) and the Everglades which had a moderate mean Zn concentration (999 ng/g ± 164 ng/g, *p* = 0.04, Tables 1 & S5). Bear Island alligator Zn concentrations were also found to be significantly different from the Zn concentrations in alligators from Yawkey, St. John’s River, Lakes Kissimmee, and Lochloosa, which are all closer to anthropogenic sources of Zn than Bear Island (LS *p* values < 0.03, Table S6) (Callender and Rice, 2000; Councell et al., 2004). Interestingly, Yawkey alligators were not observed to have significantly different Zn concentrations from alligators at all other sites, despite that mean Zn concentration being the greatest observed in alligators in this study (1255 ng/g ± 228 ng/g, Table 1).

#### 3.3.3. Transition elements

*Location* was significantly influential to the Mo concentrations observed in alligators at Lake Kissimmee (MLE *p* = 0.4). Differences were observed between Mo concentrations in alligators from Lake Trafford which had the greatest mean Mo concentration (mean Mo = 7 ng/g ± 2 ng/g, Table 1) and alligators from all other sites, except for Yawkey (LS *p* values ≤ 0.006, Table S6). The Mo concentrations observed in alligators from the Everglades (mean Mo = 3 ng/g ± 1 ng/g) and St. John’s River (mean Mo = 3 ng/g ± 1 ng/g) were also significantly different from those the Mo concentrations observed in alligators from Lake Kissimmee (mean Mo = 4 ng/g ± 2 ng/g; *p* = 0.002, *p* = 0.02, respectively) and Yawkey (mean Mo = 4 ng/g ± 1 ng/g; *p* < 0.0001, *p* = 0.002, respectively).

*Location* was influential to the Al concentrations observed in alligators at the Everglades site, which is one of the lowest mean Al concentrations observed in this study (119 ng/g ± 23 ng/g, Table 1; MLE *p* = 0.03). The LS means comparison did not reveal any significant differences between the Al concentrations at the seven study sites (Table S6). *Location* was not significant for Cu concentrations in the MLE model, and no site-specific differences were observed for Cu concentrations in the LS means comparison (Tables S5 & S6).

## 4. Discussion

The American alligators sampled in wetlands of Florida and South Carolina in this study are exposed to varying concentrations of the toxic, essential, and transition elements (Table 1). The life history characteristics of the alligator make it an attractive species for use as a sentinel, specifically their long-life spans, high site fidelity, varied diet, and their ability to live in proximity to humans (Wilkinson et al., 2016). Throughout the southeastern U.S. alligators are consumed as large game via recreational hunting and are a direct source of toxic element exposure to humans locally (FFWCC, 2015; Tipton et al., 2017a, 2017b; Smith et al., 2017). The locations sampled in this study are unique in that there are ongoing alligator monitoring programs in place which makes samples accessible for temporal studies, and the sites in Florida allow recreational hunting of alligators (FFWCC, 2015; Tipton et al., 2017a, 2017b; Smith et al., 2017). To determine how the alligator can best be utilized as a sentinel species for local trace element exposure and to understand the exposure the alligators in this study experience relative to what is known for exposure and outcomes, the measurements in this study are evaluated by comparison to existing wildlife and human data.

The comparison between wildlife and human exposure data here serves to discuss the evidence of these shared exposures in concentrations that are relevant to human health, which is critical to the evaluation of a sentinel (Rabinowitz et al., 2005a, 2009). Here, we aim to add information to the evidence gap that exists between wildlife and human health exposures, as well as reduce the separation of data that prohibits the connection of wildlife and human health outcomes (Rabinowitz et al., 2005b; Rabinowitz and Conti, 2013). This study meets the criteria for human health relevance described by Rabinowitz et al. (2005a) in that the hazard examined is relevant to human health through a shared exposure pathway, and that the exposures are discussed in relation to known health outcomes. While interspecies differences in susceptibility could not be assessed in this study, they are discussed and highlight areas of future research to further validate the alligator as a sentinel (Rabinowitz et al., 2005b; Rabinowitz and Conti, 2013).

### 4.1. Toxic elements

All four of the toxic elements (Pb, Hg, As, Cd) were observed in the alligator blood samples examined in this study at concentrations known to cause toxicity to humans and the environment (Table 1, Fig. 2) (Liu et al., 2003; Ferrario et al., 2009; de Burbure et al., 2006; Godt et al., 2006; Ruiz et al., 2010; Bini and Wahsha, 2014; Fraga, 2005; Rumbold et al., 2008).

The Hg concentrations observed in Everglades alligators are consistent with historic measurements in alligators and other taxa from that region, so the influence of *Location* at this site is not surprising (mean Hg 1364 ± 673 ng/g, Table 1) (Eisemann et al., 1997; Rumbold et al., 2001; Khan and Tansel, 2000; Frederick et al., 2005; Liu et al., 2008; Evans and Crumley, 2005; Ugarte et al., 2005; Frederick et al., 1999; Delany et al., 1988; Lance et al., 2006; Fontaine et al., 2008; McKelvey et al., 2007; Garca-Fernandez et al., 1996; Hall et al., 2006; Magley, 2017; Magley, 2018; Lemly, 2004; Raymond and Ralston, 2004). The influence of *Location* at Lakes Kissimmee (mean Hg = 393 ± 204 ng/g) and Trafford (mean Hg = 194 ± 73 ng/g) may be due to similar environmental characteristics that accelerate Hg methylation within the ecosystem, as they are closer in latitude to the Everglades than the other sites examined in this study (Fig. 1). The elevated Hg concentrations in alligators from the Everglades reported here are within the range of Hg concentrations that have been shown to cause reproductive impairment and altered mating behavior in other wetland species in the Everglades (730–3950 ng/g Hg in blood) (Frederick et al., 2005; Liu et al., 2008; Delany et al., 1988). Since the behavioral changes reported can lead to population changes in the Everglades ecosystem, these new measurements reinforce the need for continuing monitoring of Hg contamination.

The Pb blood concentrations observed in alligators in this study (range of mean concentrations = 33–906 ng/g, Table 1) are unable to be directly compared to other alligator populations, as previous studies do not report Pb in blood samples. However, the known biodistribution of Pb in alligators is such that muscle and liver tissue both have a lesser Pb concentration compared to blood (Nilsen et al., 2017b). The mean blood Pb concentrations observed in this study are within the range reported in the liver of adult alligators from other sites in Florida (35 ng/g–3400 ng/g), but lesser than the mean liver Pb concentration reported (8150 ng/g) for alligators from the Charleston, SC area (Horai et al., 2014; Campbell et al., 2010). The mean Pb concentrations in blood observed in alligators in this study are comparable to historic muscle tissue samples from alligators across Florida range from 40 ng/g to 120 ng/g Pb (Nilsen et al., 2017b; Lance et al., 2006). Using the known Pb biodistribution information we can extrapolate that the alligators samples in this study are likely to have lesser Pb concentration in their muscle and liver tissue than those previously reported, since the blood Pb concentrations from our study are comparable to previously reported muscle and liver Pb concentrations, and therefore the alligators in this study may be less exposed to Pb than alligators at other sites (Nilsen et al., 2017b). Routinely monitoring the Pb concentrations in blood samples could provide greater details regarding the baseline blood Pb concentrations for alligators in this region and will provide the opportunity to observe temporal changes in exposure.

The mean Cd concentrations observed in alligators at our study sites (range of mean concentrations = 0.7–1.2ng/g, Table 1) may appear low, but Cd is seldom detected in the blood of alligators, and is reported at concentrations in other tissues that are considered low (> 200 ng/g in liver) (Nilsen et al., 2017b; Lance et al., 2006). The Cd concentrations reported in alligators from this study are comparable to the mean Cd concentration reported for healthy unexposed human populations. Specifically, adults across the U.S. (0.4 ng/g in blood), adults in New York City (mean Cd = 0.8 ng/g), and non-smoking Nunavik Inuit adults (mean Cd = 0.6 ng/g) all have mean Cd concentrations within the range observed in our samples (McKelvey et al., 2007; Garcá-Fernández et al., 1996; Fontaine et al., 2008). These comparisons suggest that the alligators sampled in this study are not likely to be negatively impacted by Cd exposure.

The mean As concentrations observed in alligators at our study sites (23–57 ng/g, Table 1) were comparable to mean liver As concentrations reported in healthy alligators from northern Florida (33–234 ng/g), and were all greater than those associated with skin lesions in humans (16 ng/g in blood) (Bryan et al., 2007; Hall et al., 2006). While the As concentrations reported here are elevated compared to those known to elicit a negative effect in humans, they are within the range previously observed in alligator liver samples. More research needs to be done to investigate the potential detrimental effects of As exposure in long-lived predators.

### 4.2. Essential elements

The mean Se concentrations varied across the study sites, but the statistically significant locations appear to be related to their distance from Se point sources. Lake Lochloosa, Kissimmee, and St. John’s River are all in proximity to coal-fired power plants and are subject to different forms of wastewater effluent, which are known sources of Se emission (Magley, 2017, 2018; Lemly, 2004). Despite *Location* not being a significant influence on the Se concentrations in alligators at Yawkey, there is a coal-fired power plant nearby. The difference between Yawkey and the other sites in proximity to coal-fired power plants could be the specific biogeochemistry of Yawkey including the oceanic influence of that wetland region, which can aid in the dispersal of the coal effluent. The sites that are the most far removed from coal-fired power plants are Lake Trafford, Bear Island, and the Everglades. The distances from a point source of Se may be a reason for the statistical differences observed between the alligator Se concentrations at these sites and those at sites closer to power plants (Table 1).

The most interesting aspect of the reported Se concentrations is in relation to the corresponding Hg concentrations observed in alligators at each site (Table S1). The Se to Hg molar ratio has been used as an indicator of organismal response to Hg exposure, with ratios above 1 considered protective (Ralston and Raymond, 2010; Raymond and Ralston, 2004). The only site with the mean Se:Hg ratio in alligators below 1 was the Everglades (0.3, Table 1), which may correlate to the deleterious effects observed in local wildlife including reproductive impairment (Frederick et al., 2005; Frederick et al., 1999; Rumbold et al., 2008). The alligators in the Everglades could provide a useful comparison to alligators from sites with a higher Se:Hg ratio for future research.

The Zn concentrations in alligators are influenced by diet and point sources, particularly coal-fired power plants and wastewater runoff (Councell et al., 2004; Callender and Rice, 2000). Bear Island (mean Zn in alligators = 819 ± 230 ng/g, Table 1) and the Everglades (mean Zn in alligators = 999 ±164 ng/g) are the study sites that were the most far removed from point sources of Zn pollution, were influenced by *Location* in the MLE model, and had significantly different Zn concentrations from alligators at the other study sites (Tables 1, S5, S6). The greatest mean Zn concentration observed in alligators from this study was at Yawkey (1255 ± 228 ng/g), which is still far below what is considered “normal” in an urban human population (5900 ng/g in blood samples) (Khandekar et al., 1987). The mean Zn concentrations observed in this study were higher than what has been previously reported in alligator plasma from Louisiana, U.S. (430 ng/g), but since the erythrocyte proteins were not included, the studies are difficult to compare (Kushlan and Kushlan, 1980). A Zn concentration that causes toxic effects is considered anomalous, therefore the alligators sampled in this study are unlikely to experience toxicity but could provide information regarding alligator exposure to and utilization of Zn in the future.

The greater mean Zn concentration at Yawkey (1255 ± 228 ng/g, Table 1) could be due to a dietary difference at this location and the location’s proximity to a pulp mill and coal-fired power plant. Alligators at this site are known to forage in the ocean, and the dietary bioavailability of Zn is optimized by the high protein content of marine organisms (McManus and Newton, 2011). Interestingly, the Zn concentrations in Yawkey alligators were not significantly influenced by *Location*, but the Zn concentrations observed at Yawkey may have played a role in the significant influence of *Sex* across all samples for Zn concentrations in our MLE model (Table S5). Yawkey was the only site to have more female alligators sampled than males (12:3, Table 1). The skewed sex ratio at this site combined with the higher Zn concentrations could be the reason that *Sex* was a significant influencing factor for Zn concentrations in the MLE model. There may not be a true relationship with *Sex*, and instead there may be an artifact of the anthropogenic Zn influence, and different diet of alligators at Yawkey, combined with the greater proportion of females sampled. Future studies at Yawkey could investigate this relationship to determine if there is a sex-based difference in Zn accumulation in alligators.

### 4.3. Transition elements

The Al concentrations observed in the alligators from this study were not significantly different between the study sites. While *Sex* and *SVL* were influencing factors for these Al concentrations, they are likely well below the threshold that would elicit a toxic effect, as much greater Al concentrations (up to 1,500,000 ng/g) were given to juvenile doves (*Streptopelia risoria*) through dietary exposure with no effect reported (Scheuhammer, 1987). Doves are much smaller in body size than alligators, and the alligators in this study are not exposed to any known anthropogenic point sources of Al or Al contamination, the Al observed here is likely a dietary exposure and is not speculated to be problematic for this species (Scheuhammer, 1987). However, no specific reptilian or crocodilian data exists regarding Al toxicity currently and could be the focus of future studies.

The existing data for Mo concentrations in alligators or other wildlife species is sparse, and the concentrations presented in this work are the first reported for American alligators. These data can be used by other wildlife studies as a basis for comparison to determine where Mo contamination exists. As humans acquire Mo through their diet, and very few cases of Mo toxicity are known, this element may not be of concern for human populations (Vyskočil and Viau, 1999).

### 4.4. Potential as sentinel species

The American alligators sampled in this study could be used as a sentinel for sublethal exposure to toxic trace elements that are easily accessible through ongoing biomonitoring programs. The concentrations of the toxic elements (As, Hg, Pb and Cd) observed in the alligators in this study are within the range of effects previously documented in other species, but not at concentrations that may elicit the most extreme outcomes reported.

The toxic trace element exposure that local recreational hunters experience through consumption of alligators could be detrimental. Alligators recreationally caught in the Everglades are not supposed to be consumed due to high Hg contamination, but not all hunters abide by these warnings (FFWCC, 2015). There are currently no warnings regarding the consumption of alligators from Lakes Kissimmee and Trafford, which both had elevated Hg concentrations in the alligators compared to the other study sites. The Se:Hg molar ratios observed at Kissimmee and Trafford may offer some protection to the hunters consuming alligators from those locations, relative to the Se:Hg molar ratio observed in Everglades alligators. Based on the results of this study, the Pb exposure hunters may experience from alligator consumption is unpredictable, and the bioaccumulation of Cd and As could be detrimental if exposure is consistent (Hepp et al., 2017; Cardwell et al., 2013).

Alligators make attractive candidates for monitoring the biochemical changes associated with exposure. Monitoring changes in the enzymatic, epigenetic, and metabolomic changes that can be observed in blood samples will enable the identification of exposure related changes leading up to the adverse effects with known etiologies (Nilsen et al., 2016; Samuelsson et al., 2006; Lee et al., 2014; El-Demerdash, 2001). Additional studies need to be conducted to determine if the As, Hg, Pb, and Cd concentrations that alligators experience elicits the same biochemical effects observed in humans, including a comparison of the detoxification mechanisms in reptiles and mammals. Specially, differences in element assimilation efficiency through digestion, and element biotransformation into the various tissues need to be taken into consideration (Schneider et al., 2013). The bioavailability of metallothionein (MT) proteins also needs to be further investigated, since the concentration of the non-mammalian-vertebrate-specific bioavailable MT proteins in crocodilians is not related to body mass, age, or metal concentration (Buenfil-Rojas et al., 2015; Seren et al., 2014). While different than a mammal, the slower reptilian metabolism offers advantages to understanding biochemical effects of elevated exposures. Overall, the range of toxic trace element concentrations observed in this study as well as the potential for examination of biochemical changes related to chronic element exposures adds the alligator’s utility as a sentinel species.

## 5. Conclusion

The American alligators sampled and evaluated in this study are exposed to the toxic elements Hg, Pb, As, and Cd, accumulating in the coastal wetland of the southeastern U.S. The on-going monitoring programs in the southeastern Atlantic coastal region combined with robust populations and the ability to collect individuals based on their non-endangered status, make the alligator an attractive sentinel for toxic trace element exposure. As a keystone predator, the alligator provides the ability to examine both biochemical and ecosystem level changes related to the toxic element exposure, which is highly sought after when new sentinel species are described (Tabor and Aguirre, 2004). Potential changes in alligator populations due to toxic contaminant exposure can influence ecosystem dynamics, including populations of other species and trophic web interactions (Wilkinson et al., 2016; Bondavalli and Ulanowicz, 1999; Rosenblatt and Heithaus, 2011; Elsey et al., 2008; Kushlan and Kushlan, 1980; Bergeron et al., 2007). The seven sites used here enable modeling general human exposure (South Carolina and northern Florida sites), and subsistence hunting diets (Everglades sites), as well as providing important exposure information for the local reactional hunting community. The alligators in this study can be further examined to better understand the effects of toxic element exposure while preserving a vitally important coastal ecosystem.

## Supplementary Material

Supp1

## Figures and Tables

**Table 1:** The trace element concentrations (mean ± standard deviation, (range) in ng/g w/w) detected in the American alligator (*Alligator mississippiensis*) blood samples from South Carolina and Florida.

Location	Yawkey, SC	Bear Island, SC	Kissimmee, FL	Lochloosa, FL	St. Johns, FL	Trafford, FL	Everglades, FL
*n*	15	14	12	10	11	12	14
Sex ratio F:M	12:3	2:12	5:7	3:7	3:8	2:10	4:10
SVL (cm)	136 ± 21 (112–183)	119 ± 22 (80–165)	129 ± 33 (90–178)	126 ± 31 (94–180)	136 ± 20 (96–168)	121 ± 25 (90–154)	119 ± 23 (92–157)
Al^S^	53 ± 36 (15–148)	38 ± 23 (80–165)	45 ± 43 (13–171)	50 ± 30 (22 – 101)	41 ± 24 (15–83)	48 ± 34 (25–106)	66 ± 60 (18–215)
Cu	319 ± 56 (230–439)	277 ± 79 (159–394)	361 ± 155 (131–753)	372 ± 81 (225–516)	315 ± 174 (78–760)	312 ± 77 (240–514)	302 ± 104 (27–419)
Zn^S^	1255 ± 228^B^ (770–1578)	819 ± 230 (286–1225)	1203 ± 210^B^ (862–1501)	1167 ± 139^B^ (912–1413)	994 ± 312^B^ (646–1774)	902 ± 165 (727–1248)	999 ± 164 (797–1363)
Mo^S^	4 ± 1^JE^ (2–7)	4 ± 1^T^ (2–6)	4 ± 2^TJ^ (2 – 11)	4 ± 1^T^ (2–5)	3 ± 1^T^ (2–4)	7 ± 2 (5–11)	3 ± 1^TK^ (2–4)
Se	228 ± 40^EK^ (155–293)	189 ± 41^LJ^ (114–276)	174 ± 24^L^ (15–30)	289 ± 54^T^ (210–357)	213 ± 107^K^ (106–410)	199 ± 23^J^ (163–237)	160 ± 28^LJ^ (115–209)
As	33 ± 19^K^ (6–85)	31 ± 13^J^ (16–59)	23 ± 5^J^ (15–30)	23 ± 7^J^ (11–37)	57 ± 50^Y^ (10–156)	22 ± 6^J^ (11–35)	24 ± 7^J^ (12–34)
Cd	0.7 ± 0.1^EK^ (0.6–0.8)	0.7 ± 0.2 (0.3–0.9)	1.0 ± 0.1^BE^ (0.7–1.1)	1.0 ± .02^BYE^ (0.6–1.3)	1.2 ± 0.3^BY^ (0.9–1.9)	1.1 ± .02^BY^ (0.9–1.5)	1.1 ± 0.4^BL^ (0.8–2.6)
Pb	163 ± 208 (9–780)	76 ± 83 (12–266)	83 ± 168 (5–587)	488 ± 1204 (13–3891)	33 ± 48^T^ (3–167)	906 ± 73 (67–359)	464 ± 1308 (5–4872)
Hg	150 ± 49^KE^ (48–238)	118 ± 58^EK^ (44–243)	393 ± 204^EB^ (185–796)	146 ± 67^KE^ (39–251)	153 ± 49^KET^ (79–234)	194 ± 73^BLE^ (67–359)	1364 ± 673 (438–2765)
Molar ratio Se:Hg	3.9	4.1	1.1	5.0	3.5	2.6	0.3

S indicates a statistical difference for sex observed for that element using the MLE model and LS means comparison; detailed results provided in Tables S5 & S6.

Y, B, K, L, J, T, E indicate a site specific difference observed in the LS means comparison between the site with the superscript and the capital letter indicated (correlating to the site name with the corresponding first letter; i.e. Y = Yawkey, B = Bear Island, K = Kissimmee, J = St. John’s, T = Trafford, E = Everglades); details are provided in Table S6.
