# Supplementary material for: Examining toxic trace element exposure in American alligators
Source: Environ Int. Author manuscript; Available in PMC 2019 Nov 15. (PMC6857802; doi:10.1016/j.envint.2019.04.035)

Examining toxic trace element exposure in American alligators

*Supplemental Information*

**Methods**

**SAS Syntax for statistical analyses:**

- Descriptive Statistics Calculations

proc univariate data=<data file name>;

class Location;

var Al Cu Zn Mo As Se Cd Pb Hg;

run;

- T-test for Normality - Parametric Assumption Test

proc univariate data=<data file name> NORMAL;

var logAl logCu logZn logAs logSe logPb logHg logMo logCd SVL;

histogram/normal;

run;

- Analysis of Variance in the Data- Parametric Assumption Test

proc glm data=<data file name>;

class Location Sex;

model logAl logCu logZn logAs logSe logPb logHg logMo logCd =Location Sex Location*Sex;

run;

- Maximum Likelihood Estimation Model and Least Squares Mean Difference

ods graphics on;

proc lifereg data=<data file name> plots=all;

class sex location;

model (TEreported,TEmeasured <LOD)=sex location SVL__cm_ location*SVL__cm_;

lsmeans sex location / diff adjust=tukey;

run;

**Results**

**Table S1.** A list of the SRM solutions, mass fractions, uncertainties and lot numbers used in the creation of the ICP-MS and DMA-80 Calibration Curves. The certified measurement uncertainty (U) is reported in ug/g.

| **SRM Number** | **SRM Name** | **Lot #** | **Symbol** | **Mass fraction (µg/g)** | ±**U** |
| --- | --- | --- | --- | --- | --- |
| 3101a | Aluminum (Al) Standard Solution | 060502 | **Al** | 10,001 | 17 |
| 3165 | Vanadium (V) Standard Solution | 992706 | **V** | 4,860 | 20 |
| 3112a | Chromium (Cr) Standard Solution | 030730 | **Cr** | 9,922 | 25 |
| 3132 | Manganese (Mn) Standard Solution | 050429 | **Mn** | 10,000 | 20 |
| 3113 | Cobalt (Co) Standard Solution | 000630 | **Co** | 9,996 | 23 |
| 3136 | Nickel (Ni) Standard Solution | 000612 | **Ni** | 9,738 | 22 |
| 3114 | Copper (Cu) Standard Solution | 121207 | **Cu** | 10,005 | 24 |
| 3168a | Zinc (Zn) Standard Solution | 120629 | **Zn** | 10,007 | 20 |
| 3103a | Arsenic (As) Standard Solution | 100818 | **As** | 9,999 | 15 |
| 3149 | Selenium (Se) Standard Solution | 100901 | **Se** | 10,042 | 51 |
| 3145a | Rubidium (Rb) Standard Solution | 891203 | **Rb** | 10,040 | 60 |
| 3153a | Strontium (Sr) Standard Solution | 990906 | **Sr** | 9,070 | 30 |
| 3134 | Molybdenum (Mo) Standard Solution | 891307 | **Mo** | 9,990 | 30 |
| 3108 | Cadmium (Cd) Standard Solution | 060531 | **Cd** | 10,005 | 19 |
| 3161a | Tin (Sn) Standard Solution | 070330 | **Sn** | 10,010 | 21 |
| 3133 | Mercury (Hg) Standard Solution | 061204 | **Hg** | 9,954 | 53 |
| 3128 | Lead (Pb) Standard Solution | 101026 | **Pb** | 9,995 | 14 |

**Table S2.** The measured values of Seronorm as a control material throughout the analysis of the alligator blood samples. The certified measurement uncertainty (U) are reported in ng/g. Cells highlighted in yellow indicate measurement % relative standard deviation (%RSD) that is greater than the certified %RSD, both percentages are rounded to the nearest integer.

| **Seronorm** | **Al** | **V** | **Cr** | **Mn** | **Co** | **Ni** | **Cu** | **Zn** | **As** | **Se** | **Rb** | **Sr** | **Mo** | **Cd** | **Pb** |
| --- | --- | --- | --- | --- | --- | --- | --- | --- | --- | --- | --- | --- | --- | --- | --- |
| Measurement Mean | 96.99 | 6.69 | 18.97 | 42.58 | 10.45 | 11.07 | 2274.44 | 8215.01 | 32.67 | 211.85 | 1260.33 | 8.53 | 6.69 | 11.05 | 410.71 |
| Standard Deviation | 15.34 | 2.17 | 1.06 | 1.89 | 0.69 | 1.71 | 81.85 | 477.37 | 6.20 | 11.80 | 47.27 | 1.04 | 0.84 | 0.37 | 21.89 |
| %  RSD | 16% | 33% | 6% | 4% | 7% | 15% | 4% | 6% | 19% | 6% | 4% | 12% | 13% | 3% | 5% |
| Certified Values | 99.06 | 5.38 | 21.89 | 44.62 | 10.75 | 11.89 | 2330.19 | 8462.26 | 28.68 | 256.6 | 1273.58 | 14.15 | 7.08 | 11.42 | 421.70 |
| ±U | 19.81 | 1.04 | 4.43 | 8.96 | 1.13 | 2.36 | 235.85 | 622.64 | 6.89 | 51.89 | 14.15 | 0.19 | 1.42 | 1.23 | 43.40 |
| Certified  %RSD | 20% | 19% | 20% | 20% | 11% | 20% | 10% | 7% | 24% | 20% | 1% | 1% | 20% | 11% | 10% |
| LOD | 0.53 | 0.12 | 0.15 | 1.61 | 0.18 | 0.27 | 1.28 | 0.70 | 0.37 | 1.54 | 0.90 | 0.13 | 0.09 | 0.02 | 0.02 |

**Table S3.** The measured values of NIST SRM 955c Toxic Metals in Caprine Blood control material throughout the analysis of the alligator blood samples. The certified measurement uncertainty (U) are reported in ng/g.

| **Hg SRM Values for each sample group (ng/g)** | | |
| --- | --- | --- |
| **Location** | **South Carolina** | **Florida** |
| SRM 955c | Level 2 | Level 3 |
| Mean | 5.2 | 17.2 |
| Standard Deviation | 0.2 | 0.5 |
| Certified Values | 4.95 | 17.8 |
| ±U | 0.76 | 1.6 |

**Table S4.** The measured values of the 11 detected trace elements in the American alligator blood samples collected from South Carolina and Florida (ng/g).

| **Sample ID** | **Location** | **SVL (cm)** | **Sex** | **Al** | **Ni** | **Cu** | **Zn** | **As** | **Se** | **Rb** | **Mo** | **Pb** | **Hg** | **Cd** | **Capture Date** |
| --- | --- | --- | --- | --- | --- | --- | --- | --- | --- | --- | --- | --- | --- | --- | --- |
| MUSC402 | Bear Island | 136.6 | Female | 93.2 | <LOD | 284.6 | 973.9 | 38.4 | 190.2 | 385.3 | 3.83 | 114.8 | 243.3 | 0.8 | 4/11/2014 |
| MUSC406 | Bear Island | 102 | Male | 28.8 | <LOD | 360.1 | 737.0 | 25.6 | 218.5 | 432.6 | 5.25 | 36.5 | 79.1 | 0.5 | 5/1/2014 |
| MUSC409 | Bear Island | 164.6 | Male | 44.1 | <LOD | 394.1 | 741.6 | 32.3 | 192.6 | 654.3 | 3.04 | 17.8 | 64.5 | 0.8 | 5/1/2014 |
| MUSC411 | Bear Island | 87.2 | Male | 36.5 | <LOD | 362.7 | 755.4 | 59.4 | 204.0 | 627.6 | 6.12 | 86.1 | 112.5 | 0.8 | 5/1/2014 |
| MUSC415 | Bear Island | 128.5 | Female | 55.3 | <LOD | 273.5 | 1224.6 | 39.0 | 208.3 | 659.7 | 2.26 | 43.9 | 217.9 | 0.8 | 5/1/2014 |
| MUSC417 | Bear Island | 103.5 | Male | 16.1 | <LOD | 159.2 | 286.4 | 24.6 | 114.1 | 247.5 | 1.99 | 153.8 | 108.5 | 0.3 | 5/1/2014 |
| MUSC420 | Bear Island | 120 | Male | 14.4 | <LOD | 254.1 | 578.5 | 23.9 | 175.9 | 435.6 | 2.76 | 18.2 | 90.5 | 0.6 | 5/2/2014 |
| MUSC426 | Bear Island | 80.2 | Male | 22.6 | <LOD | 302.0 | 891.5 | 35.7 | 221.7 | 642.0 | 4.08 | 20.0 | 168.4 | 0.9 | 5/8/2014 |
| MUSC428 | Bear Island | 121.6 | Male | 49.9 | <LOD | 158.8 | 749.1 | 16.6 | 119.9 | 554.7 | 2.29 | 12.2 | 151.0 | 0.6 | 5/8/2014 |
| MUSC430 | Bear Island | 109.4 | Male | 44.1 | <LOD | 325.4 | 873.1 | 29.8 | 197.5 | 701.3 | 4.67 | 220.4 | 102.7 | 0.9 | 5/15/2014 |
| MUSC440 | Bear Island | 118 | Male | 23.5 | <LOD | 182.6 | 773.4 | 15.6 | 165.8 | 548.1 | 3.11 | 32.2 | 105.6 | 0.6 | 5/15/2014 |
| MUSC444 | Bear Island | 129.2 | Male | 12.9 | <LOD | 213.1 | 771.2 | 17.9 | 161.3 | 442.8 | 2.75 | 265.6 | 43.9 | 0.6 | 5/15/2014 |
| MUSC446 | Bear Island | 120.6 | Male | 67.5 | <LOD | 248.6 | 973.5 | 23.3 | 204.1 | 599.2 | 4.01 | 17.3 | 65.9 | 0.9 | 5/22/2014 |
| MUSC448 | Bear Island | 139 | Male | 27.8 | <LOD | 364.9 | 1137.4 | 51.5 | 275.9 | 666.5 | 5.62 | 27.4 | 92.4 | 0.8 | 5/22/2014 |
| Gator002 | Kissimmee | 167 | Male | 170.5 | <LOD | 321.0 | 1046.7 | 14.9 | 177.8 | 1177.9 | 3.2 | 62.2 | 795.5 | 0.7 | 3/28/2012 |
| Gator004 | Kissimmee | 177.5 | Male | 50.3 | <LOD | 753.2 | 1365.1 | 27.2 | 194.1 | 1248.1 | 4.8 | 39.1 | 792.2 | 1.0 | 3/29/2012 |
| Gator005 | Kissimmee | 106.2 | Female | 21.5 | <LOD | 389.1 | 1290.5 | 26.3 | 232.9 | 1600.8 | 3.6 | 9.2 | 331.0 | 1.0 | 3/28/2012 |
| Gator006 | Kissimmee | 109.2 | Male | 55.3 | <LOD | 221.0 | 1025.1 | 24.4 | 157.4 | 857.5 | 3.1 | 5.4 | 262.7 | 1.0 | 3/29/2012 |
| Gator009 | Kissimmee | 115.1 | Female | 12.9 | <LOD | 468.8 | 1501 | 24.4 | 150.6 | 1374.7 | 3.3 | 11.5 | 333.7 | 1.0 | 3/28/2012 |
| Gator010 | Kissimmee | 124 | Female | 57.6 | <LOD | 353.1 | 1356.8 | 16.2 | 178.3 | 1135.2 | 2.8 | 12.0 | 496.1 | <LOD | 3/29/2012 |
| Gator011 | Kissimmee | 160 | Male | 28.2 | <LOD | 131.0 | 1127.0 | 23.5 | 150.8 | 1146.9 | 2.4 | 12.5 | 412.5 | 1.0 | 3/29/2012 |
| Gator012 | Kissimmee | 142.5 | Male | 20.7 | <LOD | 233.5 | 862.6 | 29.6 | 185.1 | 991.6 | 3 | 587.2 | 305.2 | 1 | 3/28/2012 |
| Gator013 | Kissimmee | 90.4 | Female | 14.7 | <LOD | 363 | 1230 | 23.6 | 147.5 | 1198.8 | 4.2 | 18.2 | 284.2 | 1.1 | 3/28/2012 |
| Gator015 | Kissimmee | 173 | Male | 49 | <LOD | 331.7 | 886.9 | 22.4 | 165.9 | 749.5 | 2.5 | 197.7 | 300.9 | 1.1 | 3/28/2012 |
| Gator017 | Kissimmee | 96 | Male | 43.7 | <LOD | 440.8 | 1347.2 | 27.2 | 178.1 | 1998.7 | 10.5 | 26.4 | 215.7 | 1.1 | 3/28/2012 |
| Gator018 | Kissimmee | 90 | Female | 18.7 | <LOD | 320.4 | 1399.7 | 17.7 | 165 | 1231.1 | 2.7 | 8.1 | 185.3 | 0.9 | 3/28/2012 |
| Gator026 | Lochloosa | 144 | Female | 25.0 | 10.9 | 334.0 | 1323.2 | 37.0 | 247.6 | 1731.2 | 3.0 | 62.9 | 250.7 | 1.1 | 4/13/2012 |
| Gator038 | Lochloosa | 94 | Female | 49.0 | <LOD | 350.4 | 1149.9 | 21.8 | 344.6 | 1530.4 | 4.1 | 467.4 | 86.8 | 1.1 | 4/13/2012 |
| Gator046 | Lochloosa | 159 | Male | <LOD | <LOD | 334.8 | 1157.2 | 11.1 | 265.1 | 2079.3 | 1.8 | 3891.2 | 182.1 | 0.6 | 4/13/2012 |
| Gator057 | Lochloosa | 179.5 | Male | 66.3 | <LOD | 404.6 | 1031.5 | 20.1 | 267.1 | 1848.5 | 3.3 | 187.6 | 201.5 | 1.0 | 4/13/2012 |
| Gator060 | Lochloosa | 105.5 | Female | 101.4 | 5.2 | 430.3 | 1412.9 | 27.9 | 300.3 | 1587.1 | 5.2 | 17.2 | 141.1 | 1.2 | 4/13/2012 |
| Gator062 | Lochloosa | 108.8 | Male | <LOD | 5.3 | 357.8 | 1217.0 | 26.8 | 336.4 | 1639.4 | 3.8 | 15.8 | 101.9 | 1.3 | 4/13/2012 |
| Gator063 | Lochloosa | 95.8 | Male | <LOD | <LOD | 320.1 | 1192.5 | 22.8 | 335.9 | 1475.7 | 3.9 | 22.0 | 115.2 | 1.1 | 4/13/2012 |
| Gator067 | Lochloosa | 128.4 | Male | <LOD | <LOD | 450.0 | 1112.8 | 24.8 | 357.3 | 1646.2 | 4.6 | 14.4 | 116.0 | 1.0 | 4/13/2012 |
| Gator073 | Lochloosa | 93.5 | Male | 21.6 | 4.9 | 224.6 | 912.4 | 18.4 | 210.1 | 1038.7 | 3.2 | 13.2 | 38.7 | 1.0 | 4/12/2012 |
| Gator075 | Lochloosa | 151.5 | Male | 35.7 | <LOD | 515.6 | 1165.0 | 15.7 | 221.4 | 2715.1 | 2.7 | 186.0 | 227.9 | 1.0 | 4/12/2012 |
| Gator003 | St. Johns R | 138 | Male | 44.9 | <LOD | 376.8 | 1237.0 | 116.7 | 410.3 | 940.9 | 4.4 | 18.6 | 161.2 | 1.1 | 4/2/2012 |
| Gator007A | St. Johns R | 146 | Male | 76.2 | 27.7 | 77.6 | 782.6 | 39.1 | 161.4 | 651.8 | 3.5 | 19.6 | 174.0 | 1.4 | 4/2/2012 |
| Gator014 | St. Johns R | 126 | Male | 19.8 | 6.0 | 177.8 | 646.2 | 27.9 | 128.5 | 525.2 | 2.8 | 4.6 | 78.8 | 1.0 | 4/2/2012 |
| Gator016 | St. Johns R | 96 | Female | 55.8 | <LOD | 214.6 | 758.1 | 19.6 | 106.1 | 518.4 | 2.7 | 167.2 | 91.3 | 1.9 | 4/2/2012 |
| Gator019 | St. Johns R | 152.1 | Male | 25.2 | <LOD | 350.9 | 1143.9 | 109.1 | 341.5 | 1046.3 | 2.7 | 28.4 | 149.3 | 1.0 | 4/3/2012 |
| Gator020B | St. Johns R | 135.6 | Female | 16.9 | <LOD | 269.6 | 1774.1 | 81.0 | 362.0 | 1134.2 | 2.3 | 55.2 | 194.5 | 1.3 | 4/3/2012 |
| Gator022 | St. Johns R | 155.4 | Male | 14.7 | <LOD | 381.5 | 998.5 | 21.6 | 130.9 | 746.0 | 1.9 | 5.9 | 161.8 | 1.0 | 4/3/2012 |
| Gator023 | St. Johns R | 126 | Female | <LOD | <LOD | 255.6 | 1020.8 | 16.3 | 180.1 | 790.1 | 2.1 | 3.3 | 89.2 | 0.9 | 4/3/2012 |
| Gator029D | St. Johns R | 137 | Male | 36.2 | <LOD | 334.4 | 872.1 | 9.5 | 142.6 | 772.5 | 1.5 | 8.2 | 236.9 | 1.2 | 4/3/2012 |
| Gator032 | St. Johns R | 117.5 | Male | 82.9 | <LOD | 760.3 | 891.8 | 26.2 | 152.3 | 793.0 | 2.2 | 5.5 | 155.2 | 1.1 | 4/2/2012 |
| Gator033 | St. Johns R | 168.2 | Male | 34.2 | 5.6 | 270.1 | 813.3 | 155.9 | 222.9 | 610.4 | 3.3 | 49.4 | 193.2 | 1.3 | 4/3/2012 |
| Gator031 | Trafford | 99 | Male | <LOD | 7.5 | 340.6 | 1098.1 | 35.3 | 237.4 | 670.4 | 9.0 | 12.1 | 241.5 | 1.5 | 4/10/2012 |
| Gator036 | Trafford | 134 | Male | 105.5 | <LOD | 291.6 | 937.7 | 26.2 | 209.1 | 498.5 | 9.6 | 14.0 | 158.4 | 1.1 | 4/10/2012 |
| Gator039 | Trafford | 153.5 | Male | <LOD | 4.8 | 240.3 | 734.7 | 22.5 | 192.5 | 463.0 | 5.7 | 139.0 | 183.6 | 1.0 | 4/10/2012 |
| Gator041 | Trafford | 90 | Male | <LOD | <LOD | 357.0 | 1248.2 | 11.2 | 169.4 | 1192.5 | 8.3 | 11.6 | 67.4 | <LOD | 4/10/2012 |
| Gator042 | Trafford | 139.9 | Male | 24.8 | 4.8 | 270.2 | 728.5 | 23.9 | 217.6 | 602.4 | 5.6 | 4633.5 | 172.3 | 1.1 | 4/10/2012 |
| Gator044 | Trafford | 140.2 | Male | <LOD | <LOD | 260.9 | 797.5 | 16.8 | 189.1 | 460.1 | 7.7 | 20.4 | 247.0 | 0.9 | 4/10/2012 |
| Gator045 | Trafford | 109 | Male | 37.3 | 4.8 | 251.4 | 842.3 | 19.2 | 185.4 | 516.0 | 4.9 | 9.9 | 222.4 | 1.0 | 4/10/2012 |
| Gator047 | Trafford | 153 | Male | <LOD | 5.1 | 301.5 | 726.6 | 25.5 | 202.8 | 506.3 | 6.8 | 315.0 | 203.4 | 1.1 | 4/10/2012 |
| Gator049 | Trafford | 105 | Female | <LOD | <LOD | 513.6 | 1080.0 | 20.4 | 196.2 | 498.1 | 10.8 | 10.9 | 135.6 | 1.2 | 4/10/2012 |
| Gator050 | Trafford | 92 | Male | <LOD | <LOD | 264.4 | 884.7 | 23.1 | 187.7 | 691.6 | 4.7 | 9.7 | 358.6 | 1.0 | 4/10/2012 |
| Gator053 | Trafford | 93 | Female | 25.2 | <LOD | 279.6 | 890.0 | 19.0 | 162.9 | 738.2 | 5.7 | 9.6 | 134.6 | 1.1 | 4/10/2012 |
| Gator056 | Trafford | 142 | Male | 47.3 | <LOD | 377.4 | 849.3 | 20.9 | 236.2 | 557.7 | 7.0 | 5685.5 | 196.7 | 1.1 | 4/10/2012 |
| Gator030 | WCA2A | 94 | Female | <LOD | <LOD | 282.2 | 1362.7 | 33.4 | 127.0 | 859.7 | 2.1 | 6.7 | 1052.7 | 0.9 | 4/4/2012 |
| Gator043 | WCA2A | 130.2 | Male | 51.8 | <LOD | 249.2 | 796.9 | 23.0 | 165.7 | 800.4 | 2.5 | 10.3 | 2631.7 | 1.0 | 4/4/2012 |
| Gator054 | WCA2A | 140.5 | Male | 20.0 | 6.0 | 376.1 | 824.1 | 27.7 | 208.6 | 918.8 | 3.2 | 165.0 | 1880.3 | 1.2 | 4/4/2012 |
| Gator069 | WCA2A | 105 | Female | 215.4 | <LOD | 295.0 | 1220.3 | 28.6 | 142.5 | 737.4 | 3.7 | 1207.2 | 1137.5 | 1.0 | 4/4/2012 |
| Gator071 | WCA2A | 91.8 | Female | 18.2 | 4.7 | 182.4 | 1153.1 | 34.0 | 114.6 | 726.9 | 2.1 | 13.2 | 1428.7 | 1.1 | 4/5/2012 |
| Gator078 | WCA2A | 132 | Male | <LOD | <LOD | 333.6 | 1018.3 | 13.9 | 204.6 | 775.1 | 2.9 | 6.2 | 846.7 | 0.8 | 4/5/2012 |
| Gator034 | WCA3A | 94.3 | Female | <LOD | <LOD | 332.3 | 1002.9 | 22.7 | 193.5 | 698.2 | 4.2 | 6.7 | 700.4 | 1.0 | 4/5/2012 |
| Gator037 | WCA3A | 111 | Male | 60.9 | <LOD | 353.2 | 905.4 | 25.4 | 145.6 | 760.2 | 2.3 | 167.8 | 1207.6 | 1.1 | 4/4/2012 |
| Gator055 | WCA3A | 154 | Male | 82.6 | <LOD | 27.0 | 867.0 | 17.9 | 175.0 | 582.6 | 1.9 | 4871.6 | 940.1 | 2.6 | 4/5/2012 |
| Gator064 | WCA3A | 115.7 | Male | <LOD | <LOD | 384.2 | 972.3 | 17.1 | 148.1 | 1109.4 | 2.0 | 6.8 | 1066.1 | 1.0 | 4/4/2012 |
| Gator065 | WCA3A | 94 | Male | 53.2 | <LOD | 240.9 | 814.1 | 20.9 | 141.8 | 551.0 | 3.4 | 5.1 | 438.4 | 0.9 | 4/4/2012 |
| Gator066 | WCA3A | 157 | Male | 67.1 | <LOD | 419.0 | 1095.3 | 23.2 | 165.4 | 792.9 | 3.3 | 7.0 | 1554.2 | 1.1 | 4/5/2012 |
| Gator070 | WCA3A | 142 | Male | <LOD | <LOD | 395.0 | 973.7 | 12.4 | 159.2 | 745.2 | 1.8 | 9.1 | 2765.0 | 0.8 | 4/5/2012 |
| Gator077 | WCA3A | 102.1 | Male | 25.5 | 4.6 | 364.5 | 981.0 | 31.8 | 141.8 | 1442.9 | 3.6 | 6.8 | 1442.2 | 1.2 | 4/4/2012 |
| MUSC041R | Yawkey | 127 | Female | <LOD | <LOD | 438.9 | 770.2 | 5.6 | 181.0 | 343.2 | 2.91 | 95.5 | 154.9 | <LOD | 6/23/2011 |
| MUSC047 | Yawkey | 129.5 | Female | 51.7 | <LOD | 390.4 | 1078.7 | 29.8 | 243.3 | 285.7 | 5.5 | 27.4 | 146.7 | 0.7 | 6/21/2011 |
| MUSC048 | Yawkey | 113.5 | Female | 34.6 | <LOD | 332.7 | 1280.6 | 23.0 | 194.5 | 374.2 | 3.34 | 56.7 | 126.3 | 0.6 | 6/23/2011 |
| MUSC050 | Yawkey | 116.8 | Female | 28.2 | <LOD | 382.0 | 1477.0 | 33.9 | 263.9 | 411.3 | 4.45 | 256.3 | 141.2 | 0.7 | 6/24/2011 |
| MUSC051 | Yawkey | 134.62 | Female | 24.6 | <LOD | 270.5 | 1226.0 | 20.4 | 209.1 | 328.9 | 3.04 | 201.7 | 192.9 | <LOD | 6/24/2011 |
| MUSC052 | Yawkey | 129.54 | Female | 31.1 | <LOD | 278.1 | 1394.7 | 26.7 | 203.1 | 407.3 | 3.4 | 61.0 | 151.2 | 0.7 | 6/24/2011 |
| MUSC054 | Yawkey | 134.6 | Female | 109.8 | <LOD | 353.5 | 1515.7 | 21.7 | 252.2 | 457.0 | 4.51 | 199.3 | 174.1 | 0.7 | 6/24/2011 |
| MUSC055 | Yawkey | 182.9 | Male | 44.3 | <LOD | 229.9 | 1171.1 | 56.7 | 250.4 | 273.1 | 7.37 | 10.0 | 59.1 | <LOD | 6/26/2011 |
| MUSC056 | Yawkey | 120.02 | Female | 56.9 | <LOD | 317.3 | 1330.9 | 22.1 | 154.8 | 455.7 | 3.87 | 23.6 | 160.5 | 0.7 | 6/26/2011 |
| MUSC057 | Yawkey | 146.1 | Female | 36.3 | <LOD | 288.9 | 1360.3 | 21.7 | 293.2 | 234.5 | 4.78 | 147.9 | 48.2 | 0.7 | 6/28/2011 |
| MUSC058 | Yawkey | 129.5 | Female | 147.9 | <LOD | 293.0 | 1461.4 | 39.0 | 253.7 | 603.1 | 5.42 | 780.0 | 238.2 | 0.8 | 6/29/2011 |
| MUSC059 | Yawkey | 111.8 | Female | 67.5 | <LOD | 253.9 | 1577.5 | 44.4 | 201.6 | 253.9 | 5.55 | 43.5 | 207.7 | 0.7 | 6/30/2011 |
| MUSC061 | Yawkey | 134.6 | Female | 40.1 | <LOD | 331.6 | 1169.6 | 84.7 | 275.6 | 415.2 | 3.32 | 449.2 | 129.8 | 0.6 | 6/30/2011 |
| MUSC062 | Yawkey | 177 | Male | 15.3 | <LOD | 280.3 | 900.0 | 25.9 | 189.5 | 450.4 | 2.75 | 87.0 | 164.8 | <LOD | 6/30/2011 |
| MUSC063 | Yawkey | 146.05 | Male | 54.2 | <LOD | 336.9 | 1112.3 | 42.2 | 252.1 | 415.2 | 2.16 | 8.5 | 154.5 | 0.6 | 6/30/2011 |

**Table S5:** The parameter estimates from the MLE model conducted using SAS 9.4 “proc Lifereg” for left censored non-parametric data to analyze the variables influential to trace element concentration in American alligators sampled in Florida and South Carolina. The statistically significant relationships are highlighted in green.

| **Aluminum** | **Analysis of Maximum Likelihood Parameter Estimates** | | | | | | | | | |
| --- | --- | --- | --- | --- | --- | --- | --- | --- | --- | --- |
|  | **Parameter** | | **DF** | **Estimate** | **Standard Error** | **95% Confidence Limits** | | **Chi-Square** | **Pr > ChiSq** |  |
|  | **Sex** | **Female** | 1 | 0.4404 | 0.2027 | 0.0431 | 0.8376 | 4.72 | 0.0298 |  |
|  | **Sex** | **Male** | 0 | 0 | . | . | . | . | . |  |
|  | **Location** | **Bear Island** | 1 | -0.0161 | 0.2711 | -0.5475 | 0.5153 | 0 | 0.9526 |  |
|  | **Location** | **Everglades** | 1 | 0.5951 | 0.2724 | 0.0613 | 1.1289 | 4.77 | 0.0289 |  |
|  | **Location** | **Kissimmee** | 1 | 0.1411 | 0.2716 | -0.3913 | 0.6735 | 0.27 | 0.6034 |  |
|  | **Location** | **Lochloosa** | 1 | 0.0633 | 0.2931 | -0.5112 | 0.6378 | 0.05 | 0.8291 |  |
|  | **Location** | **St. Johns R** | 1 | 0.0482 | 0.2863 | -0.5129 | 0.6093 | 0.03 | 0.8662 |  |
|  | **Location** | **Trafford** | 1 | 0.373 | 0.3745 | -0.361 | 1.1069 | 0.99 | 0.3192 |  |
|  | **Location** | **Yawkey** | 0 | 0 | . | . | . | . | . |  |
|  | **SVL__cm_** |  | 1 | 0.0071 | 0.0034 | 0.0004 | 0.0138 | 4.32 | 0.0377 |  |
| **Mercury** | **Analysis of Maximum Likelihood Parameter Estimates** | | | | | | | | | |
|  | **Parameter** | | **DF** | **Estimate** | **Standard Error** | **95% Confidence Limits** | | **Chi-Square** | **Pr > ChiSq** |  |
|  | **Sex** | **Female** | 1 | 0.1015 | 0.1008 | -0.0961 | 0.2991 | 1.01 | 0.3142 |  |
|  | **Sex** | **Male** | 0 | 0 | . | . | . | . | . |  |
|  | **Location** | **Bear Island** | 1 | -0.0054 | 0.1507 | -0.3007 | 0.29 | 0 | 0.9716 |  |
|  | **Location** | **Everglades** | 1 | 2.3883 | 0.1531 | 2.0881 | 2.6884 | 243.21 | <.0001 |  |
|  | **Location** | **Kissimmee** | 1 | 0.9758 | 0.1508 | 0.6803 | 1.2713 | 41.88 | <.0001 |  |
|  | **Location** | **Lochloosa** | 1 | -0.0032 | 0.1543 | -0.3055 | 0.2991 | 0 | 0.9835 |  |
|  | **Location** | **St. Johns R** | 1 | 0.0125 | 0.1587 | -0.2986 | 0.3236 | 0.01 | 0.9372 |  |
|  | **Location** | **Trafford** | 1 | 0.507 | 0.174 | 0.166 | 0.848 | 8.49 | 0.0036 |  |
|  | **Location** | **Yawkey** | 0 | 0 | . | . | . | . | . |  |
|  | **SVL__cm_** |  | 1 | 0.0072 | 0.0018 | 0.0036 | 0.0108 | 15.24 | <.0001 |  |
| **Copper** | **Analysis of Maximum Likelihood Parameter Estimates** | | | | | | | | | |
|  | **Parameter** | | **DF** | **Estimate** | **Standard Error** | **95% Confidence Limits** | | **Chi-Square** | **Pr > ChiSq** |  |
|  | **Sex** | **Female** | 1 | -0.0354 | 0.0901 | -0.2119 | 0.1412 | 0.15 | 0.6946 |  |
|  | **Sex** | **Male** | 0 | 0 | . | . | . | . | . |  |
|  | **Location** | **Bear Island** | 1 | -0.1144 | 0.1352 | -0.3793 | 0.1505 | 0.72 | 0.3972 |  |
|  | **Location** | **Everglades** | 1 | -0.0144 | 0.1297 | -0.2686 | 0.2398 | 0.01 | 0.9117 |  |
|  | **Location** | **Kissimmee** | 1 | 0.2391 | 0.124 | -0.0039 | 0.4821 | 3.72 | 0.0537 |  |
|  | **Location** | **Lochloosa** | 1 | 0.1465 | 0.1322 | -0.1125 | 0.4056 | 1.23 | 0.2676 |  |
|  | **Location** | **St. Johns R** | 1 | 0.2061 | 0.1443 | -0.0767 | 0.4889 | 2.04 | 0.1532 |  |
|  | **Location** | **Trafford** | 1 | 0.0113 | 0.1286 | -0.2409 | 0.2634 | 0.01 | 0.9303 |  |
|  | **Location** | **Yawkey** | 0 | 0 | . | . | . | . | . |  |
|  | **SVL__cm_** |  | 1 | 0.0008 | 0.0015 | -0.0021 | 0.0037 | 0.28 | 0.5951 |  |
| **Zinc** | **Analysis of Maximum Likelihood Parameter Estimates** | | | | | | | | | |
|  | **Parameter** | | **DF** | **Estimate** | **Standard Error** | **95% Confidence Limits** | | **Chi-Square** | **Pr > ChiSq** |  |
|  | **Sex** | **Female** | 1 | 0.2227 | 0.0444 | 0.1357 | 0.3096 | 25.2 | <.0001 |  |
|  | **Sex** | **Male** | 0 | 0 | . | . | . | . | . |  |
|  | **Location** | **Bear Island** | 1 | -0.2239 | 0.0663 | -0.3539 | -0.094 | 11.4 | 0.0007 |  |
|  | **Location** | **Everglades** | 1 | -0.1341 | 0.0644 | -0.2604 | -0.0079 | 4.34 | 0.0373 |  |
|  | **Location** | **Kissimmee** | 1 | 0.043 | 0.0649 | -0.0842 | 0.1702 | 0.44 | 0.5075 |  |
|  | **Location** | **Lochloosa** | 1 | 0.0127 | 0.0703 | -0.1251 | 0.1505 | 0.03 | 0.8569 |  |
|  | **Location** | **St. Johns R** | 1 | -0.0117 | 0.0641 | -0.1373 | 0.1138 | 0.03 | 0.8546 |  |
|  | **Location** | **Trafford** | 1 | -0.1465 | 0.0779 | -0.2993 | 0.0062 | 3.53 | 0.0601 |  |
|  | **Location** | **Yawkey** | 0 | 0 | . | . | . | . | . |  |
|  | **SVL__cm_** |  | 1 | -0.0002 | 0.0008 | -0.0017 | 0.0013 | 0.06 | 0.7994 |  |
| **Arsenic** | **Analysis of Maximum Likelihood Parameter Estimates** | | | | | | | | | |
|  | **Parameter** |  | **DF** | **Estimate** | **Standard Error** | **95% Confidence Limits** | | **Chi-Square** | **Pr > ChiSq** |  |
|  | **Sex** | **Female** | 1 | 0.0436 | 0.1074 | -0.1668 | 0.254 | 0.17 | 0.6845 |  |
|  | **Sex** | **Male** | 0 | 0 | . | . | . | . | . |  |
|  | **Location** | **Bear Island** | 1 | -0.1016 | 0.175 | -0.4446 | 0.2415 | 0.34 | 0.5617 |  |
|  | **Location** | **Everglades** | 1 | -0.4302 | 0.1661 | -0.7556 | -0.1047 | 6.71 | 0.0096 |  |
|  | **Location** | **Kissimmee** | 1 | -0.5016 | 0.1648 | -0.8245 | -0.1787 | 9.27 | 0.0023 |  |
|  | **Location** | **Lochloosa** | 1 | -0.4865 | 0.1704 | -0.8206 | -0.1524 | 8.15 | 0.0043 |  |
|  | **Location** | **St. Johns R** | 1 | 0.7177 | 0.1709 | 0.3827 | 1.0528 | 17.63 | <.0001 |  |
|  | **Location** | **Trafford** | 1 | -0.5124 | 0.1788 | -0.8628 | -0.1619 | 8.21 | 0.0042 |  |
|  | **Location** | **Yawkey** | 0 | 0 | . | . | . | . | . |  |
|  | **SVL__cm_** |  | 1 | 0.0011 | 0.0019 | -0.0027 | 0.0048 | 0.3 | 0.5815 |  |
| **Selenium** | **Analysis of Maximum Likelihood Parameter Estimates** | | | | | | | | | |
|  | **Parameter** | | **DF** | **Estimate** | **Standard Error** | **95% Confidence Limits** | | **Chi-Square** | **Pr > ChiSq** |  |
|  | **Sex** | **Female** | 1 | 0.008 | 0.0564 | -0.1026 | 0.1185 | 0.02 | 0.8875 |  |
|  | **Sex** | **Male** | 0 | 0 | . | . | . | . | . |  |
|  | **Location** | **Bear Island** | 1 | -0.1363 | 0.0883 | -0.3095 | 0.0369 | 2.38 | 0.1229 |  |
|  | **Location** | **Everglades** | 1 | -0.3365 | 0.0825 | -0.4981 | -0.1748 | 16.64 | <.0001 |  |
|  | **Location** | **Kissimmee** | 1 | -0.2716 | 0.0787 | -0.426 | -0.1173 | 11.9 | 0.0006 |  |
|  | **Location** | **Lochloosa** | 1 | 0.2651 | 0.0908 | 0.0871 | 0.4431 | 8.52 | 0.0035 |  |
|  | **Location** | **St. Johns R** | 1 | 0.2086 | 0.0835 | 0.0449 | 0.3723 | 6.24 | 0.0125 |  |
|  | **Location** | **Trafford** | 1 | -0.1443 | 0.0897 | -0.32 | 0.0315 | 2.59 | 0.1077 |  |
|  | **Location** | **Yawkey** | 0 | 0 | . | . | . | . | . |  |
|  | **SVL__cm_** |  | 1 | 0.0009 | 0.0011 | -0.0012 | 0.003 | 0.7 | 0.4025 |  |
| **Molybednum** | **Analysis of Maximum Likelihood Parameter Estimates** | | | | | | | | | |
|  | **Parameter** | | **DF** | **Estimate** | **Standard Error** | **95% Confidence Limits** | | **Chi-Square** | **Pr > ChiSq** |  |
|  | **Sex** | **Female** | 1 | -0.1854 | 0.0845 | -0.3511 | -0.0198 | 4.81 | 0.0282 |  |
|  | **Sex** | **Male** | 0 | 0 | . | . | . | . | . |  |
|  | **Location** | **Bear Island** | 1 | -0.3435 | 0.1345 | -0.6072 | -0.0798 | 6.52 | 0.0107 |  |
|  | **Location** | **Everglades** | 1 | -0.6157 | 0.1267 | -0.8641 | -0.3673 | 23.61 | <.0001 |  |
|  | **Location** | **Kissimmee** | 1 | -0.1274 | 0.1417 | -0.405 | 0.1503 | 0.81 | 0.3687 |  |
|  | **Location** | **Lochloosa** | 1 | -0.3485 | 0.1325 | -0.6082 | -0.0888 | 6.92 | 0.0085 |  |
|  | **Location** | **St. Johns R** | 1 | -0.5676 | 0.1292 | -0.8209 | -0.3144 | 19.3 | <.0001 |  |
|  | **Location** | **Trafford** | 1 | 0.3269 | 0.1312 | 0.0698 | 0.584 | 6.21 | 0.0127 |  |
|  | **Location** | **Yawkey** | 0 | 0 | . | . | . | . | . |  |
|  | **SVL__cm_** |  | 1 | -0.0043 | 0.0015 | -0.0072 | -0.0014 | 8.23 | 0.0041 |  |
| **Lead** | **Analysis of Maximum Likelihood Parameter Estimates** | | | | | | | | | |
|  | **Parameter** |  | **DF** | **Estimate** | **Standard Error** | **95% Confidence Limits** | | **Chi-Square** | **Pr > ChiSq** |  |
|  | **Sex** | **Female** | 1 | 1.0426 | 0.5946 | -0.1227 | 2.2079 | 3.07 | 0.0795 |  |
|  | **Sex** | **Male** | 0 | 0 | . | . | . | . | . |  |
|  | **Location** | **Bear Island** | 1 | 0.8679 | 0.9144 | -0.9243 | 2.6601 | 0.9 | 0.3425 |  |
|  | **Location** | **Everglades** | 1 | 1.0036 | 0.74 | -0.4467 | 2.454 | 1.84 | 0.175 |  |
|  | **Location** | **Kissimmee** | 1 | -0.2684 | 0.7939 | -1.8243 | 1.2876 | 0.11 | 0.7353 |  |
|  | **Location** | **Lochloosa** | 1 | 1.1085 | 0.7852 | -0.4304 | 2.6475 | 1.99 | 0.158 |  |
|  | **Location** | **St. Johns R** | 1 | -0.98 | 0.7305 | -2.4117 | 0.4517 | 1.8 | 0.1797 |  |
|  | **Location** | **Trafford** | 1 | 1.7831 | 0.8341 | 0.1483 | 3.4178 | 4.57 | 0.0325 |  |
|  | **Location** | **Yawkey** | 0 | 0 | . | . | . | . | . |  |
|  | **SVL__cm_** |  | 1 | 0.0379 | 0.0098 | 0.0187 | 0.0571 | 14.94 | 0.0001 |  |
| **Cadmium** | **Analysis of Maximum Likelihood Parameter Estimates** | | | | | | | | | |
|  | **Parameter** | | **DF** | **Estimate** | **Standard Error** | **95% Confidence Limits** | | **Chi-Square** | **Pr > ChiSq** |  |
|  | **Sex** | **Female** | 1 | 0.0236 | 0.0696 | -0.1127 | 0.1599 | 0.12 | 0.7345 |  |
|  | **Sex** | **Male** | 0 | 0 | . | . | . | . | . |  |
|  | **Location** | **Bear Island** | 1 | 0.1157 | 0.1079 | -0.0958 | 0.3272 | 1.15 | 0.2836 |  |
|  | **Location** | **Everglades** | 1 | 0.8103 | 0.1118 | 0.5913 | 1.0294 | 52.57 | <.0001 |  |
|  | **Location** | **Kissimmee** | 1 | 0.4018 | 0.1028 | 0.2002 | 0.6034 | 15.26 | <.0001 |  |
|  | **Location** | **Lochloosa** | 1 | 0.4596 | 0.1062 | 0.2514 | 0.6679 | 18.72 | <.0001 |  |
|  | **Location** | **St. Johns R** | 1 | 0.6515 | 0.0988 | 0.4578 | 0.8451 | 43.48 | <.0001 |  |
|  | **Location** | **Trafford** | 1 | 0.5256 | 0.1139 | 0.3023 | 0.7489 | 21.29 | <.0001 |  |
|  | **Location** | **Yawkey** | 0 | 0 | . | . | . | . | . |  |
|  | **SVL__cm_** |  | 1 | 0.0001 | 0.0011 | -0.0022 | 0.0023 | 0 | 0.9606 |  |

**Table S6:** The Least Squares Mean Difference comparison results for the American alligator blood trace element concentrations with a Tukey-Kramer post-hoc correction for multiple comparisons (SAS 9.4, Cary, NC). The statistically significant relationships are highlighted in green.

| **Arsenic (As)** | **Differences of Sex Least Squares Means** | | | | | | |
| --- | --- | --- | --- | --- | --- | --- | --- |
|  | **Adjustment for Multiple Comparisons: Tukey-Kramer** | | | | | | |
|  | **Sex** | **_Sex** | **Estimate** | **Standard Error** | **z Value** | **Pr > \|z\|** | **Adj P** |
|  | **Female** | **Male** | 0.04362 | 0.1074 | 0.41 | 0.6845 | 0.684 |
|  | **Differences of Location Least Squares Means** | | | | | | |
|  | **Adjustment for Multiple Comparisons: Tukey-Kramer** | | | | | | |
|  | **Location** | **_Location** | **Estimate** | **Standard Error** | **z Value** | **Pr > \|z\|** | **Adj P** |
|  | **Bear Island** | **Everglades** | 0.3286 | 0.1527 | 2.15 | 0.0314 | 0.3223 |
|  | **Bear Island** | **Kissimmee** | 0.4 | 0.161 | 2.48 | 0.013 | 0.1649 |
|  | **Bear Island** | **Lochloosa** | 0.3849 | 0.17 | 2.26 | 0.0236 | 0.2618 |
|  | **Bear Island** | **St. Johns R** | -0.8193 | 0.1783 | -4.6 | <.0001 | <.0001 |
|  | **Bear Island** | **Trafford** | 0.4108 | 0.1565 | 2.63 | 0.0087 | 0.1184 |
|  | **Bear Island** | **Yawkey** | -0.1016 | 0.175 | -0.58 | 0.5617 | 0.9974 |
|  | **Everglades** | **Kissimmee** | 0.07145 | 0.1587 | 0.45 | 0.6526 | 0.9994 |
|  | **Everglades** | **Lochloosa** | 0.05634 | 0.1658 | 0.34 | 0.7341 | 0.9999 |
|  | **Everglades** | **St. Johns R** | -1.1479 | 0.1806 | -6.36 | <.0001 | <.0001 |
|  | **Everglades** | **Trafford** | 0.08222 | 0.1596 | 0.52 | 0.6064 | 0.9987 |
|  | **Everglades** | **Yawkey** | -0.4302 | 0.1661 | -2.59 | 0.0096 | 0.1288 |
|  | **Kissimmee** | **Lochloosa** | -0.01511 | 0.1702 | -0.09 | 0.9293 | 1 |
|  | **Kissimmee** | **St. Johns R** | -1.2193 | 0.1755 | -6.95 | <.0001 | <.0001 |
|  | **Kissimmee** | **Trafford** | 0.01078 | 0.1653 | 0.07 | 0.948 | 1 |
|  | **Kissimmee** | **Yawkey** | -0.5016 | 0.1648 | -3.04 | 0.0023 | 0.0377 |
|  | **Lochloosa** | **St. Johns R** | -1.2042 | 0.1857 | -6.48 | <.0001 | <.0001 |
|  | **Lochloosa** | **Trafford** | 0.02589 | 0.1749 | 0.15 | 0.8824 | 1 |
|  | **Lochloosa** | **Yawkey** | -0.4865 | 0.1704 | -2.85 | 0.0043 | 0.0652 |
|  | **St. Johns R** | **Trafford** | 1.2301 | 0.1801 | 6.83 | <.0001 | <.0001 |
|  | **St. Johns R** | **Yawkey** | 0.7177 | 0.1709 | 4.2 | <.0001 | 0.0005 |
|  | **Trafford** | **Yawkey** | -0.5124 | 0.1788 | -2.87 | 0.0042 | 0.0632 |
| **Lead (Pb)** | **Differences of Sex Least Squares Means** | | | | | | |
|  | **Adjustment for Multiple Comparisons: Tukey-Kramer** | | | | | | |
|  | **Sex** | **_Sex** | **Estimate** | **Standard Error** | **z Value** | **Pr > \|z\|** | **Adj P** |
|  | **Female** | **Male** | 1.0426 | 0.5946 | 1.75 | 0.0795 | 0.0795 |
|  | **Differences of Location Least Squares Means** | | | | | | |
|  | **Adjustment for Multiple Comparisons: Tukey-Kramer** | | | | | | |
|  | **Location** | **_Location** | **Estimate** | **Standard Error** | **z Value** | **Pr > \|z\|** | **Adj P** |
|  | **Bear Island** | **Everglades** | -0.1357 | 0.7023 | -0.19 | 0.8468 | 1 |
|  | **Bear Island** | **Kissimmee** | 1.1363 | 0.7266 | 1.56 | 0.1179 | 0.7056 |
|  | **Bear Island** | **Lochloosa** | -0.2406 | 0.7625 | -0.32 | 0.7523 | 0.9999 |
|  | **Bear Island** | **St. Johns R** | 1.8479 | 0.771 | 2.4 | 0.0165 | 0.1997 |
|  | **Bear Island** | **Trafford** | -0.9152 | 0.7125 | -1.28 | 0.199 | 0.8594 |
|  | **Bear Island** | **Yawkey** | 0.8679 | 0.9144 | 0.95 | 0.3425 | 0.9644 |
|  | **Everglades** | **Kissimmee** | 1.272 | 0.6793 | 1.87 | 0.0611 | 0.4986 |
|  | **Everglades** | **Lochloosa** | -0.1049 | 0.6962 | -0.15 | 0.8802 | 1 |
|  | **Everglades** | **St. Johns R** | 1.9836 | 0.6858 | 2.89 | 0.0038 | 0.0586 |
|  | **Everglades** | **Trafford** | -0.7794 | 0.6755 | -1.15 | 0.2485 | 0.9111 |
|  | **Everglades** | **Yawkey** | 1.0036 | 0.74 | 1.36 | 0.175 | 0.825 |
|  | **Kissimmee** | **Lochloosa** | -1.3769 | 0.7349 | -1.87 | 0.061 | 0.4979 |
|  | **Kissimmee** | **St. Johns R** | 0.7116 | 0.7299 | 0.97 | 0.3296 | 0.9594 |
|  | **Kissimmee** | **Trafford** | -2.0514 | 0.7108 | -2.89 | 0.0039 | 0.0597 |
|  | **Kissimmee** | **Yawkey** | -0.2684 | 0.7939 | -0.34 | 0.7353 | 0.9999 |
|  | **Lochloosa** | **St. Johns R** | 2.0885 | 0.7407 | 2.82 | 0.0048 | 0.0716 |
|  | **Lochloosa** | **Trafford** | -0.6745 | 0.7339 | -0.92 | 0.358 | 0.9696 |
|  | **Lochloosa** | **Yawkey** | 1.1085 | 0.7852 | 1.41 | 0.158 | 0.7957 |
|  | **St. Johns R** | **Trafford** | -2.763 | 0.7447 | -3.71 | 0.0002 | 0.0039 |
|  | **St. Johns R** | **Yawkey** | -0.98 | 0.7305 | -1.34 | 0.1797 | 0.8323 |
|  | **Trafford** | **Yawkey** | 1.7831 | 0.8341 | 2.14 | 0.0325 | 0.3303 |
| **Mercury (Hg)** | **Differences of Sex Least Squares Means** | | | | | | |
|  | **Adjustment for Multiple Comparisons: Tukey-Kramer** | | | | | | |
|  | **Sex** | **_Sex** | **Estimate** | **Standard Error** | **z Value** | **Pr > \|z\|** | **Adj P** |
|  | **Female** | **Male** | 0.1015 | 0.1008 | 1.01 | 0.3142 | 0.3142 |
|  | **Differences of Location Least Squares Means** | | | | | | |
|  | **Adjustment for Multiple Comparisons: Tukey-Kramer** | | | | | | |
|  | **Location** | **_Location** | **Estimate** | **Standard Error** | **z Value** | **Pr > \|z\|** | **Adj P** |
|  | **Bear Island** | **Everglades** | -2.3936 | 0.1383 | -17.31 | <.0001 | <.0001 |
|  | **Bear Island** | **Kissimmee** | -0.9811 | 0.155 | -6.33 | <.0001 | <.0001 |
|  | **Bear Island** | **Lochloosa** | -0.00217 | 0.1562 | -0.01 | 0.9889 | 1 |
|  | **Bear Island** | **St. Johns R** | -0.01787 | 0.1527 | -0.12 | 0.9069 | 1 |
|  | **Bear Island** | **Trafford** | -0.5123 | 0.1445 | -3.55 | 0.0004 | 0.0072 |
|  | **Bear Island** | **Yawkey** | -0.00537 | 0.1507 | -0.04 | 0.9716 | 1 |
|  | **Everglades** | **Kissimmee** | 1.4125 | 0.1471 | 9.6 | <.0001 | <.0001 |
|  | **Everglades** | **Lochloosa** | 2.3915 | 0.1517 | 15.76 | <.0001 | <.0001 |
|  | **Everglades** | **St. Johns R** | 2.3758 | 0.1458 | 16.29 | <.0001 | <.0001 |
|  | **Everglades** | **Trafford** | 1.8813 | 0.1466 | 12.83 | <.0001 | <.0001 |
|  | **Everglades** | **Yawkey** | 2.3883 | 0.1531 | 15.6 | <.0001 | <.0001 |
|  | **Kissimmee** | **Lochloosa** | 0.979 | 0.1536 | 6.37 | <.0001 | <.0001 |
|  | **Kissimmee** | **St. Johns R** | 0.9633 | 0.1506 | 6.39 | <.0001 | <.0001 |
|  | **Kissimmee** | **Trafford** | 0.4688 | 0.1695 | 2.77 | 0.0057 | 0.0827 |
|  | **Kissimmee** | **Yawkey** | 0.9758 | 0.1508 | 6.47 | <.0001 | <.0001 |
|  | **Lochloosa** | **St. Johns R** | -0.0157 | 0.1572 | -0.1 | 0.9205 | 1 |
|  | **Lochloosa** | **Trafford** | -0.5102 | 0.1704 | -2.99 | 0.0028 | 0.0439 |
|  | **Lochloosa** | **Yawkey** | -0.0032 | 0.1543 | -0.02 | 0.9835 | 1 |
|  | **St. Johns R** | **Trafford** | -0.4945 | 0.1632 | -3.03 | 0.0024 | 0.0394 |
|  | **St. Johns R** | **Yawkey** | 0.0125 | 0.1587 | 0.08 | 0.9372 | 1 |
|  | **Trafford** | **Yawkey** | 0.507 | 0.174 | 2.91 | 0.0036 | 0.0552 |
| **Cadmium (Cd)** | **Differences of Sex Least Squares Means** | | | | | | |
|  | **Adjustment for Multiple Comparisons: Tukey-Kramer** | | | | | | |
|  | **Sex** | **_Sex** | **Estimate** | **Standard Error** | **z Value** | **Pr > \|z\|** | **Adj P** |
|  | **Female** | **Male** | 0.02359 | 0.06955 | 0.34 | 0.7345 | 0.7345 |
|  | **Differences of Location Least Squares Means** | | | | | | |
|  | **Adjustment for Multiple Comparisons: Tukey-Kramer** | | | | | | |
|  | **Location** | **_Location** | **Estimate** | **Standard Error** | **z Value** | **Pr > \|z\|** | **Adj P** |
|  | **Bear Island** | **Everglades** | -0.6946 | 0.09615 | -7.22 | <.0001 | <.0001 |
|  | **Bear Island** | **Kissimmee** | -0.2861 | 0.09116 | -3.14 | 0.0017 | 0.0283 |
|  | **Bear Island** | **Lochloosa** | -0.3439 | 0.09242 | -3.72 | 0.0002 | 0.0037 |
|  | **Bear Island** | **St. Johns R** | -0.5358 | 0.09413 | -5.69 | <.0001 | <.0001 |
|  | **Bear Island** | **Trafford** | -0.4099 | 0.09105 | -4.5 | <.0001 | 0.0001 |
|  | **Bear Island** | **Yawkey** | 0.1157 | 0.1079 | 1.07 | 0.2836 | 0.9363 |
|  | **Everglades** | **Kissimmee** | 0.4086 | 0.099 | 4.13 | <.0001 | 0.0007 |
|  | **Everglades** | **Lochloosa** | 0.3507 | 0.1029 | 3.41 | 0.0007 | 0.0117 |
|  | **Everglades** | **St. Johns R** | 0.1589 | 0.1001 | 1.59 | 0.1123 | 0.6901 |
|  | **Everglades** | **Trafford** | 0.2847 | 0.1023 | 2.78 | 0.0054 | 0.0791 |
|  | **Everglades** | **Yawkey** | 0.8103 | 0.1118 | 7.25 | <.0001 | <.0001 |
|  | **Kissimmee** | **Lochloosa** | -0.05784 | 0.09641 | -0.6 | 0.5486 | 0.9968 |
|  | **Kissimmee** | **St. Johns R** | -0.2497 | 0.09515 | -2.62 | 0.0087 | 0.1188 |
|  | **Kissimmee** | **Trafford** | -0.1238 | 0.09811 | -1.26 | 0.2069 | 0.8692 |
|  | **Kissimmee** | **Yawkey** | 0.4018 | 0.1028 | 3.91 | <.0001 | 0.0018 |
|  | **Lochloosa** | **St. Johns R** | -0.1918 | 0.09757 | -1.97 | 0.0493 | 0.4365 |
|  | **Lochloosa** | **Trafford** | -0.06599 | 0.09958 | -0.66 | 0.5075 | 0.9945 |
|  | **Lochloosa** | **Yawkey** | 0.4596 | 0.1062 | 4.33 | <.0001 | 0.0003 |
|  | **St. Johns R** | **Trafford** | 0.1258 | 0.1012 | 1.24 | 0.2137 | 0.8771 |
|  | **St. Johns R** | **Yawkey** | 0.6515 | 0.0988 | 6.59 | <.0001 | <.0001 |
|  | **Trafford** | **Yawkey** | 0.5256 | 0.1139 | 4.61 | <.0001 | <.0001 |
| **Selenium (Se)** | **Differences of Sex Least Squares Means** | | | | | | |
|  | **Adjustment for Multiple Comparisons: Tukey-Kramer** | | | | | | |
|  | **Sex** | **_Sex** | **Estimate** | **Standard Error** | **z Value** | **Pr > \|z\|** | **Adj P** |
|  | **Female** | **Male** | 0.007978 | 0.05641 | 0.14 | 0.8875 | 0.8875 |
|  | **Differences of Location Least Squares Means** | | | | | | |
|  | **Adjustment for Multiple Comparisons: Tukey-Kramer** | | | | | | |
|  | **Location** | **_Location** | **Estimate** | **Standard Error** | **z Value** | **Pr > \|z\|** | **Adj P** |
|  | **Bear Island** | **Everglades** | 0.2002 | 0.07357 | 2.72 | 0.0065 | 0.0931 |
|  | **Bear Island** | **Kissimmee** | 0.1353 | 0.08035 | 1.68 | 0.0922 | 0.6268 |
|  | **Bear Island** | **Lochloosa** | -0.4014 | 0.08034 | -5 | <.0001 | <.0001 |
|  | **Bear Island** | **St. Johns R** | -0.3449 | 0.08353 | -4.13 | <.0001 | 0.0007 |
|  | **Bear Island** | **Trafford** | 0.007963 | 0.07587 | 0.1 | 0.9164 | 1 |
|  | **Bear Island** | **Yawkey** | -0.1363 | 0.08835 | -1.54 | 0.1229 | 0.7188 |
|  | **Everglades** | **Kissimmee** | -0.06484 | 0.07762 | -0.84 | 0.4036 | 0.9813 |
|  | **Everglades** | **Lochloosa** | -0.6015 | 0.08045 | -7.48 | <.0001 | <.0001 |
|  | **Everglades** | **St. Johns R** | -0.5451 | 0.08082 | -6.74 | <.0001 | <.0001 |
|  | **Everglades** | **Trafford** | -0.1922 | 0.07621 | -2.52 | 0.0117 | 0.1514 |
|  | **Everglades** | **Yawkey** | -0.3365 | 0.08248 | -4.08 | <.0001 | 0.0009 |
|  | **Kissimmee** | **Lochloosa** | -0.5367 | 0.08477 | -6.33 | <.0001 | <.0001 |
|  | **Kissimmee** | **St. Johns R** | -0.4802 | 0.08357 | -5.75 | <.0001 | <.0001 |
|  | **Kissimmee** | **Trafford** | -0.1274 | 0.08263 | -1.54 | 0.1232 | 0.7197 |
|  | **Kissimmee** | **Yawkey** | -0.2716 | 0.07875 | -3.45 | 0.0006 | 0.0101 |
|  | **Lochloosa** | **St. Johns R** | 0.05647 | 0.08977 | 0.63 | 0.5293 | 0.9959 |
|  | **Lochloosa** | **Trafford** | 0.4093 | 0.08326 | 4.92 | <.0001 | <.0001 |
|  | **Lochloosa** | **Yawkey** | 0.2651 | 0.09082 | 2.92 | 0.0035 | 0.0544 |
|  | **St. Johns R** | **Trafford** | 0.3529 | 0.0859 | 4.11 | <.0001 | 0.0008 |
|  | **St. Johns R** | **Yawkey** | 0.2086 | 0.08353 | 2.5 | 0.0125 | 0.1601 |
|  | **Trafford** | **Yawkey** | -0.1443 | 0.08968 | -1.61 | 0.1077 | 0.6768 |
| **Zinc (Zn)** | **Differences of Sex Least Squares Means** | | | | | | |
|  | **Adjustment for Multiple Comparisons: Tukey-Kramer** | | | | | | |
|  | **Sex** | **_Sex** | **Estimate** | **Standard Error** | **z Value** | **Pr > \|z\|** | **Adj P** |
|  | **Female** | **Male** | 0.2227 | 0.04436 | 5.02 | <.0001 | <.0001 |
|  | **Differences of Location Least Squares Means** | | | | | | |
|  | **Adjustment for Multiple Comparisons: Tukey-Kramer** | | | | | | |
|  | **Location** | **_Location** | **Estimate** | **Standard Error** | **z Value** | **Pr > \|z\|** | **Adj P** |
|  | **Bear Island** | **Everglades** | -0.08979 | 0.05997 | -1.5 | 0.1343 | 0.7466 |
|  | **Bear Island** | **Kissimmee** | -0.2669 | 0.06273 | -4.26 | <.0001 | 0.0004 |
|  | **Bear Island** | **Lochloosa** | -0.2366 | 0.06552 | -3.61 | 0.0003 | 0.0056 |
|  | **Bear Island** | **St. Johns R** | -0.2122 | 0.06725 | -3.16 | 0.0016 | 0.0268 |
|  | **Bear Island** | **Trafford** | -0.07742 | 0.06517 | -1.19 | 0.2349 | 0.899 |
|  | **Bear Island** | **Yawkey** | -0.2239 | 0.06632 | -3.38 | 0.0007 | 0.013 |
|  | **Everglades** | **Kissimmee** | -0.1772 | 0.06249 | -2.83 | 0.0046 | 0.0687 |
|  | **Everglades** | **Lochloosa** | -0.1468 | 0.06532 | -2.25 | 0.0246 | 0.27 |
|  | **Everglades** | **St. Johns R** | -0.1224 | 0.06698 | -1.83 | 0.0676 | 0.5292 |
|  | **Everglades** | **Trafford** | 0.01238 | 0.06602 | 0.19 | 0.8513 | 1 |
|  | **Everglades** | **Yawkey** | -0.1341 | 0.06443 | -2.08 | 0.0373 | 0.3632 |
|  | **Kissimmee** | **Lochloosa** | 0.03033 | 0.06781 | 0.45 | 0.6547 | 0.9994 |
|  | **Kissimmee** | **St. Johns R** | 0.05474 | 0.06727 | 0.81 | 0.4158 | 0.9837 |
|  | **Kissimmee** | **Trafford** | 0.1895 | 0.07082 | 2.68 | 0.0074 | 0.1042 |
|  | **Kissimmee** | **Yawkey** | 0.04301 | 0.06489 | 0.66 | 0.5075 | 0.9945 |
|  | **Lochloosa** | **St. Johns R** | 0.02441 | 0.07218 | 0.34 | 0.7352 | 0.9999 |
|  | **Lochloosa** | **Trafford** | 0.1592 | 0.07153 | 2.23 | 0.026 | 0.2816 |
|  | **Lochloosa** | **Yawkey** | 0.01268 | 0.0703 | 0.18 | 0.8569 | 1 |
|  | **St. Johns R** | **Trafford** | 0.1348 | 0.07769 | 1.73 | 0.0828 | 0.5924 |
|  | **St. Johns R** | **Yawkey** | -0.01173 | 0.06405 | -0.18 | 0.8546 | 1 |
|  | **Trafford** | **Yawkey** | -0.1465 | 0.07793 | -1.88 | 0.0601 | 0.4936 |
| **Molybdenum (Mo)** | **Differences of Sex Least Squares Means** | | | | | | |
|  | **Adjustment for Multiple Comparisons: Tukey-Kramer** | | | | | | |
|  | **Sex** | **_Sex** | **Estimate** | **Standard Error** | **z Value** | **Pr > \|z\|** | **Adj P** |
|  | **Female** | **Male** | -0.1854 | 0.08451 | -2.19 | 0.0282 | 0.0282 |
|  | **Differences of Location Least Squares Means** | | | | | | |
|  | **Adjustment for Multiple Comparisons: Tukey-Kramer** | | | | | | |
|  | **Location** | **_Location** | **Estimate** | **Standard Error** | **z Value** | **Pr > \|z\|** | **Adj P** |
|  | **Bear Island** | **Everglades** | 0.2722 | 0.1176 | 2.31 | 0.0207 | 0.2371 |
|  | **Bear Island** | **Kissimmee** | -0.2162 | 0.121 | -1.79 | 0.0741 | 0.5576 |
|  | **Bear Island** | **Lochloosa** | 0.005001 | 0.1307 | 0.04 | 0.9695 | 1 |
|  | **Bear Island** | **St. Johns R** | 0.2241 | 0.1276 | 1.76 | 0.0792 | 0.5784 |
|  | **Bear Island** | **Trafford** | -0.6704 | 0.1213 | -5.53 | <.0001 | <.0001 |
|  | **Bear Island** | **Yawkey** | -0.3435 | 0.1345 | -2.55 | 0.0107 | 0.1406 |
|  | **Everglades** | **Kissimmee** | -0.4883 | 0.1236 | -3.95 | <.0001 | 0.0015 |
|  | **Everglades** | **Lochloosa** | -0.2672 | 0.1269 | -2.11 | 0.0353 | 0.3494 |
|  | **Everglades** | **St. Johns R** | -0.04808 | 0.1273 | -0.38 | 0.7057 | 0.9998 |
|  | **Everglades** | **Trafford** | -0.9426 | 0.1203 | -7.84 | <.0001 | <.0001 |
|  | **Everglades** | **Yawkey** | -0.6157 | 0.1267 | -4.86 | <.0001 | <.0001 |
|  | **Kissimmee** | **Lochloosa** | 0.2212 | 0.1367 | 1.62 | 0.1056 | 0.6706 |
|  | **Kissimmee** | **St. Johns R** | 0.4403 | 0.1346 | 3.27 | 0.0011 | 0.0185 |
|  | **Kissimmee** | **Trafford** | -0.4542 | 0.1272 | -3.57 | 0.0004 | 0.0066 |
|  | **Kissimmee** | **Yawkey** | -0.1274 | 0.1417 | -0.9 | 0.3687 | 0.9728 |
|  | **Lochloosa** | **St. Johns R** | 0.2191 | 0.1369 | 1.6 | 0.1094 | 0.6818 |
|  | **Lochloosa** | **Trafford** | -0.6754 | 0.1316 | -5.13 | <.0001 | <.0001 |
|  | **Lochloosa** | **Yawkey** | -0.3485 | 0.1325 | -2.63 | 0.0085 | 0.1169 |
|  | **St. Johns R** | **Trafford** | -0.8945 | 0.1303 | -6.87 | <.0001 | <.0001 |
|  | **St. Johns R** | **Yawkey** | -0.5676 | 0.1292 | -4.39 | <.0001 | 0.0002 |
|  | **Trafford** | **Yawkey** | 0.3269 | 0.1312 | 2.49 | 0.0127 | 0.1619 |
| **Aluminum (Al)** | **Differences of Sex Least Squares Means** | | | | | | |
|  | **Adjustment for Multiple Comparisons: Tukey-Kramer** | | | | | | |
|  | **Sex** | **_Sex** | **Estimate** | **Standard Error** | **z Value** | **Pr > \|z\|** | **Adj P** |
|  | **Female** | **Male** | 0.4404 | 0.2027 | 2.17 | 0.0298 | 0.0298 |
|  | **Differences of Location Least Squares Means** | | | | | | |
|  | **Adjustment for Multiple Comparisons: Tukey-Kramer** | | | | | | |
|  | **Location** | **_Location** | **Estimate** | **Standard Error** | **z Value** | **Pr > \|z\|** | **Adj P** |
|  | **Bear Island** | **Everglades** | -0.6112 | 0.2562 | -2.39 | 0.017 | 0.2044 |
|  | **Bear Island** | **Kissimmee** | -0.1572 | 0.2479 | -0.63 | 0.5259 | 0.9957 |
|  | **Bear Island** | **Lochloosa** | -0.0794 | 0.3014 | -0.26 | 0.7922 | 1 |
|  | **Bear Island** | **St. Johns R** | -0.06433 | 0.2454 | -0.26 | 0.7932 | 1 |
|  | **Bear Island** | **Trafford** | -0.3891 | 0.3359 | -1.16 | 0.2468 | 0.9096 |
|  | **Bear Island** | **Yawkey** | -0.01611 | 0.2711 | -0.06 | 0.9526 | 1 |
|  | **Everglades** | **Kissimmee** | 0.454 | 0.2752 | 1.65 | 0.0991 | 0.6499 |
|  | **Everglades** | **Lochloosa** | 0.5318 | 0.3123 | 1.7 | 0.0886 | 0.6141 |
|  | **Everglades** | **St. Johns R** | 0.5469 | 0.2767 | 1.98 | 0.0481 | 0.4298 |
|  | **Everglades** | **Trafford** | 0.2221 | 0.3626 | 0.61 | 0.5402 | 0.9964 |
|  | **Everglades** | **Yawkey** | 0.5951 | 0.2724 | 2.18 | 0.0289 | 0.3036 |
|  | **Kissimmee** | **Lochloosa** | 0.07783 | 0.3137 | 0.25 | 0.804 | 1 |
|  | **Kissimmee** | **St. Johns R** | 0.0929 | 0.2571 | 0.36 | 0.7179 | 0.9998 |
|  | **Kissimmee** | **Trafford** | -0.2318 | 0.3437 | -0.67 | 0.4999 | 0.994 |
|  | **Kissimmee** | **Yawkey** | 0.1411 | 0.2716 | 0.52 | 0.6034 | 0.9986 |
|  | **Lochloosa** | **St. Johns R** | 0.01507 | 0.3185 | 0.05 | 0.9623 | 1 |
|  | **Lochloosa** | **Trafford** | -0.3097 | 0.3972 | -0.78 | 0.4356 | 0.9869 |
|  | **Lochloosa** | **Yawkey** | 0.06329 | 0.2931 | 0.22 | 0.8291 | 1 |
|  | **St. Johns R** | **Trafford** | -0.3247 | 0.3476 | -0.93 | 0.3502 | 0.9671 |
|  | **St. Johns R** | **Yawkey** | 0.04822 | 0.2863 | 0.17 | 0.8662 | 1 |
|  | **Trafford** | **Yawkey** | 0.373 | 0.3745 | 1 | 0.3192 | 0.955 |
| **Copper (Cu)** | **Differences of Sex Least Squares Means** | | | | | | |
|  | **Adjustment for Multiple Comparisons: Tukey-Kramer** | | | | | | |
|  | **Sex** | **_Sex** | **Estimate** | **Standard Error** | **z Value** | **Pr > \|z\|** | **Adj P** |
|  | **Female** | **Male** | -0.03536 | 0.09006 | -0.39 | 0.6946 | 0.6946 |
|  | **Differences of Location Least Squares Means** | | | | | | |
|  | **Adjustment for Multiple Comparisons: Tukey-Kramer** | | | | | | |
|  | **Location** | **_Location** | **Estimate** | **Standard Error** | **z Value** | **Pr > \|z\|** | **Adj P** |
|  | **Bear Island** | **Everglades** | -0.1 | 0.1129 | -0.89 | 0.3757 | 0.9748 |
|  | **Bear Island** | **Kissimmee** | -0.3535 | 0.127 | -2.78 | 0.0054 | 0.0789 |
|  | **Bear Island** | **Lochloosa** | -0.2609 | 0.1265 | -2.06 | 0.0391 | 0.3751 |
|  | **Bear Island** | **St. Johns R** | -0.3205 | 0.1221 | -2.62 | 0.0087 | 0.1184 |
|  | **Bear Island** | **Trafford** | -0.1257 | 0.1199 | -1.05 | 0.2947 | 0.9427 |
|  | **Bear Island** | **Yawkey** | -0.1144 | 0.1352 | -0.85 | 0.3972 | 0.98 |
|  | **Everglades** | **Kissimmee** | -0.2535 | 0.1238 | -2.05 | 0.0406 | 0.3848 |
|  | **Everglades** | **Lochloosa** | -0.1609 | 0.1246 | -1.29 | 0.1966 | 0.8562 |
|  | **Everglades** | **St. Johns R** | -0.2205 | 0.1228 | -1.8 | 0.0726 | 0.5511 |
|  | **Everglades** | **Trafford** | -0.02564 | 0.1188 | -0.22 | 0.8291 | 1 |
|  | **Everglades** | **Yawkey** | -0.01438 | 0.1297 | -0.11 | 0.9117 | 1 |
|  | **Kissimmee** | **Lochloosa** | 0.09258 | 0.1297 | 0.71 | 0.4752 | 0.9918 |
|  | **Kissimmee** | **St. Johns R** | 0.03302 | 0.1331 | 0.25 | 0.8041 | 1 |
|  | **Kissimmee** | **Trafford** | 0.2278 | 0.1287 | 1.77 | 0.0767 | 0.5685 |
|  | **Kissimmee** | **Yawkey** | 0.2391 | 0.124 | 1.93 | 0.0537 | 0.461 |
|  | **Lochloosa** | **St. Johns R** | -0.05956 | 0.1353 | -0.44 | 0.6597 | 0.9995 |
|  | **Lochloosa** | **Trafford** | 0.1353 | 0.1297 | 1.04 | 0.2971 | 0.9441 |
|  | **Lochloosa** | **Yawkey** | 0.1465 | 0.1322 | 1.11 | 0.2676 | 0.9257 |
|  | **St. Johns R** | **Trafford** | 0.1948 | 0.1292 | 1.51 | 0.1316 | 0.7404 |
|  | **St. Johns R** | **Yawkey** | 0.2061 | 0.1443 | 1.43 | 0.1532 | 0.7867 |
|  | **Trafford** | **Yawkey** | 0.01126 | 0.1286 | 0.09 | 0.9303 | 1 |

**Figure S1:** The Sex and SVL (cm) variables compared to the mass fraction of Hg, Pb, Cd, and As observed in American alligators (*Alligator mississippiensis*) sampled in Florida and South Carolina.


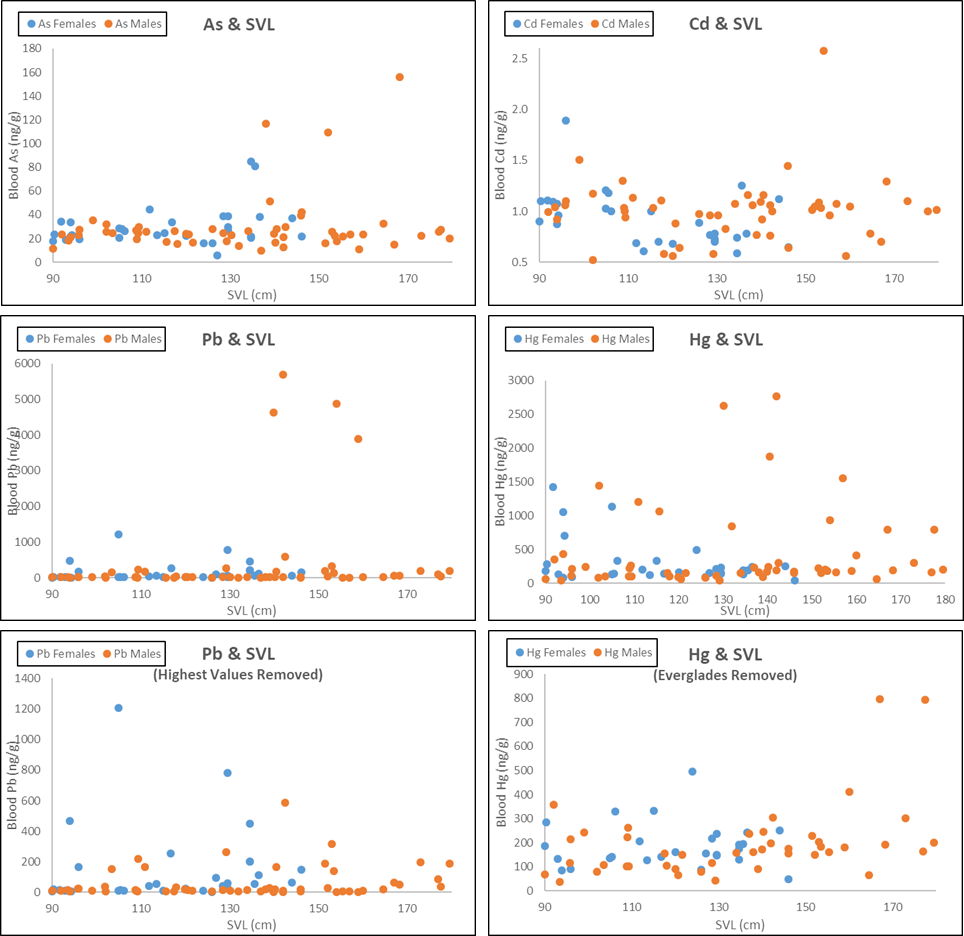

Supplement: Supp1 [file NIHMS1541694-supplement-Supp1.docx]
